# Treatment of Status Epilepticus after Traumatic Brain Injury Using an Antiseizure Drug Combined with a Tissue Recovery Enhancer Revealed by Systems Biology

**DOI:** 10.3390/ijms241814049

**Published:** 2023-09-13

**Authors:** Natallie Kajevu, Anssi Lipponen, Pedro Andrade, Ivette Bañuelos, Noora Puhakka, Elina Hämäläinen, Teemu Natunen, Mikko Hiltunen, Asla Pitkänen

**Affiliations:** 1 A. I. Virtanen Institute for Molecular Sciences, University of Eastern Finland, P.O. Box 1627, 70211 Kuopio, Finland; 2 Expert Microbiology Unit, Finnish Institute for Health and Welfare, P.O. Box 95, 70701 Kuopio, Finland; 3 Institute of Biomedicine, University of Eastern Finland, P.O. Box 1627, 70211 Kuopio, Finland

**Keywords:** biomarker, electroencephalography, fluid-percussion injury, gene expression, in silico, neuronal culture, neuroprotection, oxidative stress, post-traumatic epilepsy, seizure

## Abstract

We tested a hypothesis that in silico-discovered compounds targeting traumatic brain injury (TBI)-induced transcriptomics dysregulations will mitigate TBI-induced molecular pathology and augment the effect of co-administered antiseizure treatment, thereby alleviating functional impairment. In silico bioinformatic analysis revealed five compounds substantially affecting TBI-induced transcriptomics regulation, including calpain inhibitor, chlorpromazine, geldanamycin, tranylcypromine, and trichostatin A (TSA). In vitro exposure of neuronal-BV2-microglial co-cultures to compounds revealed that TSA had the best overall neuroprotective, antioxidative, and anti-inflammatory effects. In vivo assessment in a rat TBI model revealed that TSA as a monotherapy (1 mg/kg/d) or in combination with the antiseizure drug levetiracetam (LEV 150 mg/kg/d) mildly mitigated the increase in plasma levels of the neurofilament subunit pNF-H and cortical lesion area. The percentage of rats with seizures during 0–72 h post-injury was reduced in the following order: TBI-vehicle 80%, TBI-TSA (1 mg/kg) 86%, TBI-LEV (54 mg/kg) 50%, TBI-LEV (150 mg/kg) 40% (*p* < 0.05 vs. TBI-vehicle), and TBI-LEV (150 mg/kg) combined with TSA (1 mg/kg) 30% (*p* < 0.05). Cumulative seizure duration was reduced in the following order: TBI-vehicle 727 ± 688 s, TBI-TSA 898 ± 937 s, TBI-LEV (54 mg/kg) 358 ± 715 s, TBI-LEV (150 mg/kg) 42 ± 64 (*p* < 0.05 vs. TBI-vehicle), and TBI-LEV (150 mg/kg) combined with TSA (1 mg/kg) 109 ± 282 s (*p* < 0.05). This first preclinical intervention study on post-TBI acute seizures shows that a combination therapy with the tissue recovery enhancer TSA and LEV was safe but exhibited no clear benefit over LEV monotherapy on antiseizure efficacy. A longer follow-up is needed to confirm the possible beneficial effects of LEV monotherapy and combination therapy with TSA on chronic post-TBI structural and functional outcomes, including epileptogenesis.

## 1. Introduction

Traumatic brain injury (TBI) caused by an external mechanical force results in primary and secondary brain pathologies [1] that may lead to behavioral [2] psychiatric [3,4], and cognitive disabilities [5,6], as well as post-traumatic epilepsy [7,8]. TBI-induced gene network dysregulation, which orchestrates the development and progression of secondary brain damage, occurs via multiple molecular pathways [9], including those regulating apoptosis [10,11], neuroinflammation [12,13], and oxidative stress [9,11]. Importantly, at the acute phase of injury, these pathologies progress in parallel with TBI-induced epileptiform activity and status epilepticus (SE) in at least 30% of patients with severe TBI, thereby worsening the secondary injury by increasing intracranial pressure, cerebral edema, and metabolic crisis [14,15,16,17,18,19]. We previously reported post-TBI SE in 70–80% of rats after severe lateral fluid-percussion–induced (FPI) TBI [20]. Pre-clinical hypothesis-driven monotherapy studies have identified a large number of compounds that alter, halt, or even reverse post-TBI complications [21,22]. Whether or not co-mitigation of the molecular tissue pathology and seizure activity presents a more efficacious disease-modifying approach for disrupting the progression of secondary damage and improving the functional outcome, however, is unclear [11,23].

Attempts to apply tissue-pathology-derived omics data for the discovery of pathology-mitigating compounds have shown great promise for the treatment of a wide range of complex diseases, including amyloid-load-decreasing compounds for Alzheimer’s disease [24], antiseizure compounds for temporal lobe epilepsy [25], and tumor-growth-reducing drugs for glioblastoma [26]. We previously developed a non-hypothesis driven transcriptomics-based in silico to in vitro to in vivo pipeline that provides an unbiased approach for identifying compounds that can be repurposed to mitigate the progression of TBI-induced brain pathologies [27,28,29].

Current treatment for SE resulting from different etiologies, including TBI, is symptomatic. That is, the treatment goal is to suppress epileptiform activity and seizures rather than to mitigate the underlying pathology. Previous experimental and clinical studies, however, suggest that SE-related epileptiform activity itself, continuing for about 30 min, can cause brain damage [17,30,31]. After TBI, SE and related damage can progress for hours to days in parallel to secondary tissue damage triggered by TBI. We hypothesized that a compound regulating the gene networks related to neurodegeneration, neuroinflammation, and oxidative stress in silico and in vitro would reduce the corresponding cellular pathologies in vivo. Thus, we expected that early treatment using a “tissue repair enhancer” in combination with the antiseizure drug levetiracetam (LEV), a commonly used drug to treat early post-TBI seizures in humans, would improve the antiseizure efficacy of LEV monotherapy and mitigate structural damage better than LEV alone.

## 2. Results

### 2.1. Mortality and Exclusions

***Acute (<48 h) mortality and exclusions***. In the TBI group, acute mortality was 0% in cohort 1 and 12% (4/34) in cohort 2. Rats with a broken dura (*n* = 2), disconnection of injury cap (*n* = 1), or long (58 min) righting reflex time (*n* = 1) were excluded.

**Figure 1:**
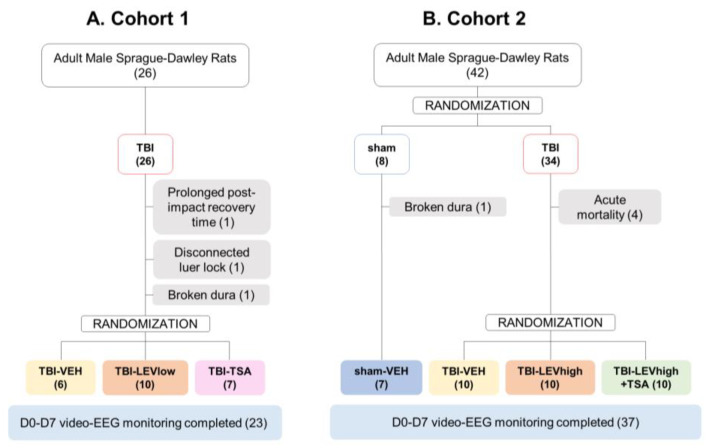
**Randomization and study flow**. A total of 68 rats were randomized into the sham-operated experimental control (*n* = 8) or TBI (*n* = 60) groups. Animals assigned to the TBI group were further randomized to either vehicle, LEV, TSA, or LEV + TSA treatments. (**A**) The final analysis Cohort 1 included 6 rats in the TBI-VEH, 10 in the TBI-LEV, and 7 in the TBI-TSA groups. (**B**) The final analysis Cohort 2 included 7 rats in the Sham-VEH, 10 in the TBI-VEH, 10 in the TBI-LEV, and 10 in the TBI-LEV + TSA groups. Reasons for exclusions are shown in grey shading (number of rats is in parentheses). **Abbreviations**: D, day; EEG, electroencephalography; LEV, levetiracetam; TBI, traumatic brain injury; TSA, trichostatin A; VEH, vehicle.

***Follow-up mortality and exclusions.*** There was no follow-up mortality. Reasons for exclusions are indicated in Figure 1.

### 2.2. Compound Selection for In Vitro Validation

The 40 compounds with the highest absolute concordance scores are listed in Supplementary Table S1. After a literature-based evaluation of the pharmacodynamic/kinetic properties, including half-life, brain–blood barrier penetration, solubility, availability, and price, five compounds, including calpain inhibitor I, chlorpromazine, geldanamycin, tranylcypromine, and trichostatin A, were chosen for further in vitro validation.

Further support for compound selection was obtained from the gene network analysis (compound-sig vs. TBI-sig; Supplementary Tables S2–S4). Particularly, at 32 h post-TBI, gene network analysis revealed the regulation of cell death and inflammatory/immune pathways by the five compounds selected (Supplementary Table S2).

### 2.3. Effect of Compounds on Neuronal Viability, Oxidative Stress, and Neuroinflammation In Vitro

#### 2.3.1. Neuronal Viability

**Figure 2:**
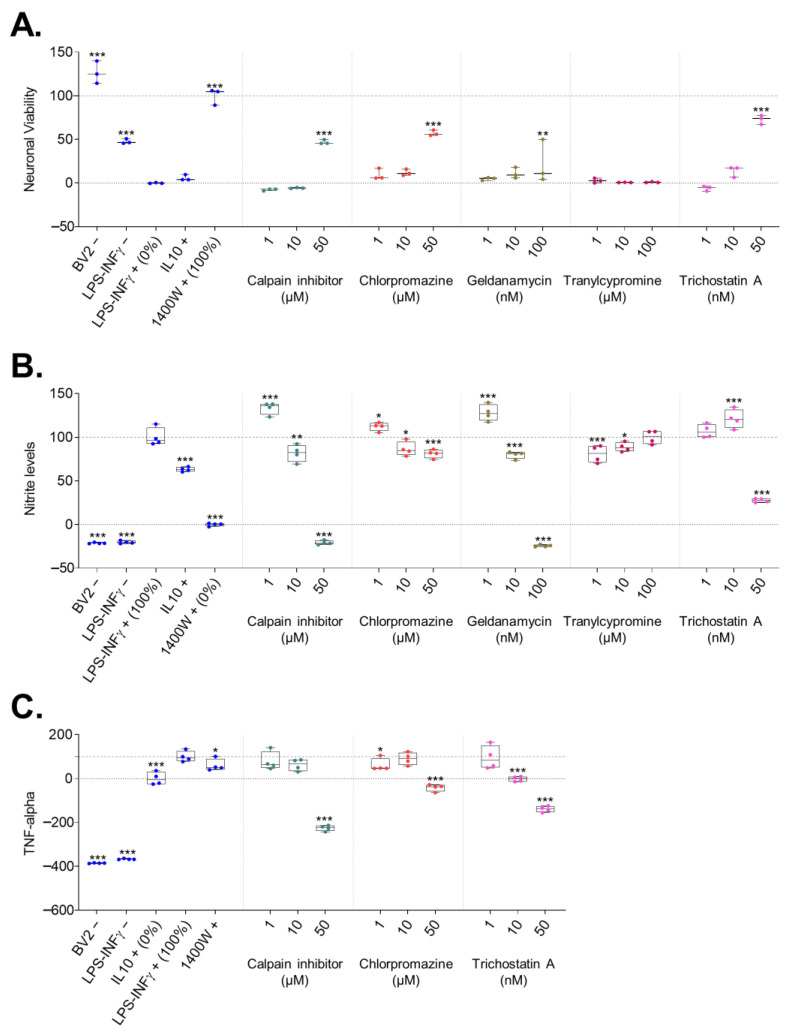
**Effect of in silico-identified drugs on tissue biomarkers of neuroprotection**, **oxidative stress**, **and neuroinflammation.** (**A**) ***Neuronal viability***. For a comparative viability analysis, neuronal survival in LPS/IFNγ+-treated neuronal–microglial co-cultures was set to 0% and that in co-cultures treated with iNOS inhibitor 1400 W (positive treatment control) to 100%. **Trichostatin A** at a 50 nM concentration increased neuronal survival from 0% to 73% (*p* < 0.001 compared with LPS/IFNγ+). The neuroprotective effect of TSA was almost as good as that of the positive control 1400 W (73% vs. 100%, *p* < 0.001). Also, **chlorpromazine** at a 50-µM concentration increased neuronal survival from 0% to 57% (*p* < 0.001) and **calpain inhibitor** at a 50-µM concentration increased neuronal survival from 0% to 47% (*p* < 0.001). **Geldanamycin** and **tranylcypromine** showed no consistent neuroprotection. **IL10** had no effect on neuronal viability. Note that in untreated co-cultures without neuroinflammation (LPS/IFNγ−), neuronal viability was 38% of that in untreated BV2 neuronal cultures (*p* < 0.001) and 48% of that in 1400 W treated inflammation-exposed co-cultures (*p* < 0.001). Wells with cortical neurons only (BV2− cells without LPS/IFNγ+ exposure) showed the greatest viability. (**B**) ***Nitrite levels.*** For a comparative viability analysis, nitrite levels in LPS/IFNγ+-treated neuronal–microglial co-culture medium were set to 100% and that in co-culture medium treated with iNOS inhibitor 1400 W (positive treatment control) was set to 0%. **Trichostatin A** at a 50 nM concentration reduced the LPS/IFNγ+-induced nitrite release from 100% to 28% (*p* < 0.001). At a 10 nM concentration, there was a slight increase in nitrite levels to 121% (*p* < 0.01). **Calpain inhibitor** at 50 µM reduced nitrite release from 100% to −21% (*p* < 0.001). **Chlorpromazine** at 50 µM reduced nitrite levels to 81% (*p* < 0.001). At 10 µM, **tranylcypromine** reduced nitrite levels from 100% to 89% (*p* < 0.05) and at 1 µM, to 81% (*p* < 0.001). Interestingly, tranylcypromine at 100 µM had no effect (*p* > 0.05). **Geldanamycin** at 100 nM concentrations resulted in low nitrite levels, likely due to the cell death that was visualized in microscopic images (data not shown). **IL10** had a mild effect on the LPS/IFNγ+-induced increase in nitrite levels, reducing it to 63% (*p* < 0.001). Note that neuron-only (BV2−) and LPS/IFNγ−neuronal–microglial co-cultures were not exposed to neuroinflammation, and thus showed no nitrite release to the culture medium. (**C**) ***TNFα levels.*** For a comparative viability analysis, TNFα levels in LPS/IFNγ+-treated neuronal–microglial co-culture medium were set to 100% and that in co-culture medium treated with TNFα inhibitor, IL10 (positive treatment control) to 0%. **Trichostatin A** at 10 nM concentration reduced TNFα levels from 100% to −2% (*p* < 0.001) and at 50 nM to −139% (*p* < 0.001), being better than the positive control. **Calpain inhibitor** at 50 µM reduced TNFα levels from 100% to −226% (*p* < 0.001). **Chlorpromazine** at 50 µM reduced TNFα levels from 100% to −21% (*p* < 0.001). The iNOS inhibitor, at **1400 W**, reduced TNFα levels from 100% to 61% (*p* < 0.05). **Abbreviations**: BV2−, cortical neuronal cultures without BV2-microglial cells; IL10+, co-cultures treated with positive control anti-inflammatory incytokine, interleukin 10; LPS/IFNγ−, co-culture wells without lipopolysaccharides and interferon γ-induced neuroinflammation; LPS/IFNγ+, wells with lipopolysaccharides and interferon γ-induced neuroinflammation (untreated controls); TNFα, tumor necrosis factor α; 1400 W+, co-cultures treated with positive control, inducible nitric oxide synthase (iNOS) inhibitor, 1400 W hydrochloride. **Statistical significance**: ***, *p* < 0.001; **, *p* < 0.01; *, *p* < 0.05 compared with LPS + IFNγ+ (linear regression in R). The number of independent experiments in panel A was three and in panels B-C was four. Data are presented as box plots with whiskers (minimum and maximum; box: interquartile range; line: median). Each dot shows an experimental value.

A neuronal viability assay was carried out to assess the effect of drugs on neuronal survival. Neuronal viability of untreated co-cultures exposed to neuroinflammation (LPS/IFNγ+) was set to 0%. Neuronal viability in a culture treated with a positive control, 1400 W, was set to 100%. Efficacy of the test drugs was expressed as a percentage of neuronal viability in the culture medium compared with that in untreated culture. Data were obtained from three independent experiments and are summarized in Figure 2A.

**Controls**. Neuronal viability in control co-cultures (LPS/IFNγ−) was 38% (*p* < 0.001) of that in neuron-only (BV2−) cultures and 48% of that in 1400 W treated LPS/IFNγ-exposed co-cultures (*p* < 0.001). IL10 had no effect on neuronal survival (6% compared with 1400 W, *p* > 0.05).

**Drugs.** Trichostatin A at a 50 nM concentration reduced LPS/IFNγ+-induced cell death, with neuronal viability 73% of that in 1400 W treated cultures (*p* < 0.001 compared with untreated LPS/IFNγ+ co-cultures). Also, 50 µM chlorpromazine (57% of that in 1400 W, *p* < 0.001 compared with untreated LPS/IFNγ+ co-cultures) and 50 µM calpain inhibitor i (47% of that in 1400 W, *p* < 0.001 compared with untreated LPS/IFNγ+ co-cultures) improved neuronal viability.

In addition to the neuronal viability assay, we performed a qualitative visual analysis of microscope images captured from drug-treated LPS/IFNγ+ exposed co-cultures at 24 h after induction of neuroinflammation (data not shown). Microscope images reflected the results obtained from the neuronal viability assay and demonstrated that 50 nM trichostatin A, 50 µM chlorpromazine, and 50 µM calpain inhibitor i promoted the survival of cortical neurons, whereas 100 nM geldanamycin was toxic to both cortical neurons and BV2−microglia cells.

#### 2.3.2. Nitrite Levels

The nitrite concentration in the untreated LPS/IFNγ+ exposed co-culture medium was set to 100%. The nitrite level in the culture treated with iNOS inhibitor 1400 W (positive control) was set to 0% (*p* < 0.001). Efficacy of the test drugs was expressed as a percentage of nitrite in the culture medium compared with that in untreated culture. Data were obtained from four independent experiments and are summarized in Figure 2B.

**Controls**. As expected, medium from the negative control cultures (BV2− and LPS/IFNγ−) had even lower nitrite levels (−21% and −20%, respectively) than medium from the 1400 W treated cultures (both *p* < 0.001 compared with that in untreated culture). IL10 reduced the LPS/IFNγ+-induced increase in nitrite levels (to 63% compared with untreated culture, *p* < 0.001).

**Drugs**. Trichostatin A at a 50 nM concentration reduced the LPS/IFNγ+-induced increase in nitrite levels in the co-culture medium (to 28% of that in untreated culture, *p* < 0.001). Nitrite levels were also reduced by treatment with 10 µM (to 87%, *p* < 0.05) or 50 µM (to 81%, *p* < 0.001) chlorpromazine, as well as by treatment with 10 µM (to 82%, *p* < 0.01) or 50 µM (to −21%, *p* < 0.001) calpain inhibitor i. Tranylcypromine reduced nitrite levels at concentrations of 1 µM (to 81%, *p* < 0.001) and 10 µM (to 89%, *p* < 0.05), whereas 100 µM tranylcypromine had no effect (*p* > 0.05). Co-cultures treated with 10 nM or 100 nM of geldanamycin had reduced levels of nitrite (*p* < 0.05), which likely related to the cell death visualized in microscopic images rather than to the antioxidant efficacy of geldanamycin.

#### 2.3.3. TNFα Levels

A TNFα ELISA was carried out to assess the effect of the drugs on neuroinflammation.

The TNFα level in the medium from untreated LPS/IFNγ+ exposed co-culture was set to 100%. The TNFα level in the culture after treatment with the anti-inflammatory cytokine IL10 (positive control) was set to 0% (*p* < 0.001). The efficacy of the test drugs was expressed as a percentage of TNFα in the culture medium compared with that in untreated culture. Data were obtained from four independent experiments and are summarized in Figure 2C.

**Controls**. Negative controls (BV2and LPS/IFNγ−) had even lower levels of TNFα than the IL10-treated cultures (−385% and −367%, respectively; *p* < 0.001 compared with that in untreated culture). The iNOS inhibitor, at 1400 W, reduced TNFα levels to 61% (*p* < 0.05).

**Drugs**. Trichostatin A at a 50 nM concentration had an even greater TNFα-level-reducing effect than IL10 (to −139%, *p* < 0.001). Also, 10 nM trichostatin exhibited an anti-inflammatory effect (to −2%, *p* < 0.001). Chlorpromazine at a 50 µM concentration (to −41%, *p* < 0.001) and calpain inhibitor i at 50 µM (to −226%, *p* < 0.001) also reduced the release of TNFα into the co-culture medium. The TNFα ELISA was not carried out for geldanamycin and tranylcypromine as they showed no effect on neuronal viability or nitrite levels.

### 2.4. Compound Selection for In Vivo Validation

The selection of a compound for in vivo validation was based on the scoring criteria summarized in Supplementary Table S5 and analysis of the post-exposure microscope images for toxicity (data not shown).

The scoring criteria revealed that calpain inhibitor i had the highest total score (Table 1). However, trichostatin A (TSA) was selected for in vivo validation, as it had the highest neuronal viability score. Also, post-exposure microscopic images revealed greater neuronal survival in TSA-treated cultures than in calpain inhibitor-treated cultures.

### 2.5. Effect of TSA on In Vivo Outcome Measures

#### 2.5.1. Preliminary Data from Cohort 1

***Plasma pNF-H.*** Plasma pNF-H was measured in the preliminary lower-dose cohort (Cohort 1) only. Data are summarized in Supplementary Figure S1A.

In the TBI-VEH group (*n* = 3), the average plasma pNF-H level was 7394 ± 592 pg/mL (median 7626 pg/mL, range 6721–7835 pg/mL). In the TBI-TSA group (*n* = 7), average pNF-H levels (6073 ± 1695 pg/mL, median 5914 pg/mL, range 3185–8533 pg/mL) were comparable to those in the TBI-VEH group (*p* > 0.05). Cohen’s delta was 0.885, however, indicating a moderate favorable effect size. Also, in the TBI-LEV group (*n* = 10), average pNF-H levels (5183 ± 3256 pg/mL, median 5696 pg/mL, range 449–9286 pg/mL) were comparable to those in the TBI-VEH (2183 ± 166 pg/mL, median 2211 pg/mL, range 2005–2333 pg/mL) (*p* > 0.05). Cohen’s delta suggested an unfavorable effect size for LEV (−1.018). In 3 of 10 rats in the TBI-LEV groups, however, the pNF-H levels were <2000 pg/mL, which were the lowest values measured.

No association was found between the total number of seizures preceding the plasma sampling or the time from the last seizure to blood sampling (*p* > 0.05).

**Cortical *lesion* area**. Cortical lesion areas were measured from unfolded cortical maps as described earlier [32]. Data are summarized in Supplementary Figure S1B–D.

On D14 after injury, the average cortical lesion area was comparable between the TBI-VEH (25.9 ± 3.0 mm^2^, median 25.0 mm^2^, range 23.1–29.2 mm^2^, *n* = 3) and TBI-TSA (22.8 ± 5.0 mm^2^, median 21.4 mm^2^, range 17.5–30.7 mm^2^, *n* = 5) groups. Cohen’s delta was 0.696, however, indicating a mild favorable effect size. Also, in the TBI-LEV (low-dose) group, the average cortical lesion area (27.1 ± 13.7 mm^2^, median 29.9 mm^2^, range 4.8–50.9 mm^2^, n = 8) was comparable to that in the corresponding TBI-VEH group (20.4 ± 4.4 mm^2^, median 21.9 mm^2^, range 15.4–23.9 mm^2^, *n* = 3) (*p* > 0.05). Cohen’s delta was −0.551, suggesting a mild unfavorable effect size for LEV. Interestingly, plasma pNF-H levels correlated with the total cortical lesion area (r = 0.558, r^2^ = 0.31, *p* < 0.05). Images from two representative animals with pNF-H measurements are shown in Supplementary Figure S1C,D.

On D180, when the greatest progression of cortical damage had already occurred [33], the average cortical lesion area was comparable between the TBI-VEH (11.2 ± 9.9 mm^2^, median 6.4 mm^2^, range 2.3–27.9 mm^2^, *n* = 10), TBI-LEV (high-dose) (15.7 ± 12.0 mm^2^, median 13.9 mm^2^, range 2.9–38.5 mm^2^, *n* = 9) and TBI-LEV + TSA (18.4 ± 13.9 mm^2^, median 13.8 mm^2^, range 5.1–41.2 mm^2^, *n* = 10) groups (*p* > 0.05).

#### 2.5.2. Acute Seizures

Occurrence and phenotype of acute post-injury seizures during 0–72 h were comparable in Cohorts 1 and 2 (*p* > 0.05) and comparable to our earlier description of the model [20]. Therefore, the data from the vehicle-treated animals were combined for further analysis.

##### Percentage of Rats with Acute Seizures during the First 72 h

**Figure 3:**
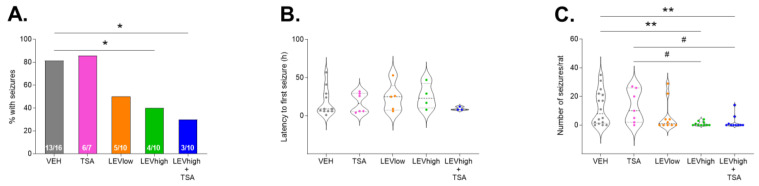
**Prevalence**, **latency**, **and number of electrographic seizures in different treatment groups during the first 72 h post-TBI.** (**A**) Percentage of rats with early seizures (*y*-axis) during the first 72 h post-TBI varied between treatment groups (*p* < 0.05 Fisher’s exact test). The number within each bar indicates the number of rats that had seizures during the first 72 h post-TBI. Note the reduction in the percentage of rats with seizures in the ***TBI-LEVhigh*** and ***TBI-LEVhigh + TSA*** groups compared with the ***TBI-VEH*** group (*p* < 0.05, Fisher’s exact test). (**B**) The latency to the first electrographic early seizure (*y*-axis) was comparable between the treatment groups (*x*-axis) (*p* > 0.05, Kruskal–Wallis). (**C**) The total number of early seizures (*y*-axis) in different treatment groups varied during the 72 h post-TBI follow-up (*x*-axis) (*p* < 0.05, Kruskal–Wallis). The number of early seizures in the ***TBI-LEVhigh*** and ***TBI-LEVhigh + TSA*** groups was lower than that in the ***TBI-VEH*** group (*p* < 0.01, Kruskal–Wallis followed by Mann–Whitney U post hoc test). Each dot in panels (**B**,**C**) represents data from 1 animal. Dashed lines in violin plots show the quartiles and the middle line is the median. **Abbreviations:** LEV, levetiracetam; TBI, traumatic brain injury; TSA, trichostatin A; VEH, vehicle. **Statistical significance:** *, *p* < 0.05 compared with the TBI-VEH group; **, *p* < 0.01 compared with the ***TBI-VEH*** group; #, *p* < 0.05 compared with the ***TBI-TSA*** group.

During the first 72 h after TBI, 81% (13/16) of the rats in the TBI-VEH group exhibited electrographic seizures. Fisher’s exact test revealed treatment effects on seizure occurrence. The percentage of rats with electrographic seizures in the TBI-LEVhigh (40%, 4/10) and TBI-LEVhigh + TSA (30%, 3/10) groups was reduced compared with that in the TBI-VEH group (*p* < 0.05) (Figure 3A). When all rats treated with a higher dose of LEV were pooled (TBI-LEVhigh and TBI-LEVhigh +TSA), the antiseizure effect became even clearer compared with that in the TBI-vehicle group (35% (7/20) vs. 81% (13/16), *p* < 0.01). Co-administration of TSA did not strengthen the LEV efficacy (*p* > 0.05). The lower dose of LEV showed a trend toward a reduced percentage of animals with seizures compared with that in the TBI-VEH group (50% (5/10) vs. 81% (13/16), *p* > 0.05). The prevalence of rats with seizures in the TBI-TSA monotherapy group (86%, 6/7) was comparable to that in the TBI-VEH group (*p* > 0.05) (Figure 3A).

##### Latency to the First Acute Seizure

In rats with acute seizures in the TBI-VEH group, the average latency to the first post-impact seizure was 17.7 ± 19.9 h (median 9.0 h, Figure 3B). Contrary to our expectation, LEV did not delay the latency to the first seizure in animals with seizures (lower dose 23.8 ± 18.7 h, higher dose 25.3 ± 16.9 h, both *p* > 0.05 compared with the vehicle group). Also, TSA monotherapy (17.2 ± 13.1 h) or LEV + TSA combination therapy (9.3 ± 2.3 h) did not affect the latency to the first acute seizure (both *p* > 0.05 compared with the vehicle).

##### Number of Acute Electrographic Seizures during the First 72 h

Data are summarized in Figure 3C and Supplementary Table S6.

In the TBI-VEH group *(n* = 16), the average number of seizures was 12.4 ± 12.7. The Kruskal–Wallis test revealed treatment effects on the number of electrographic seizures between groups (*p* < 0.01). The number of seizures was reduced in both the TBI-LEVhigh (*n* = 10, 6.0 ± 10.5) and TBI-LEVhigh + TSA (*n* = 10, 2.1 ± 4.6) groups compared with that in the TBI-VEH group (both *p* < 0.01). Also, the number of seizures showed a trend toward reduction in the TBI-LEVlow group (*n* = 10, 6.0 ± 10.5, Cohen’s delta 0.521, *p* > 0.05). Instead, TSA monotherapy (*n* = 7) showed no effect on the number of acute seizures (12.9 ± 11.4, *p* > 0.05).

**Figure 4:**
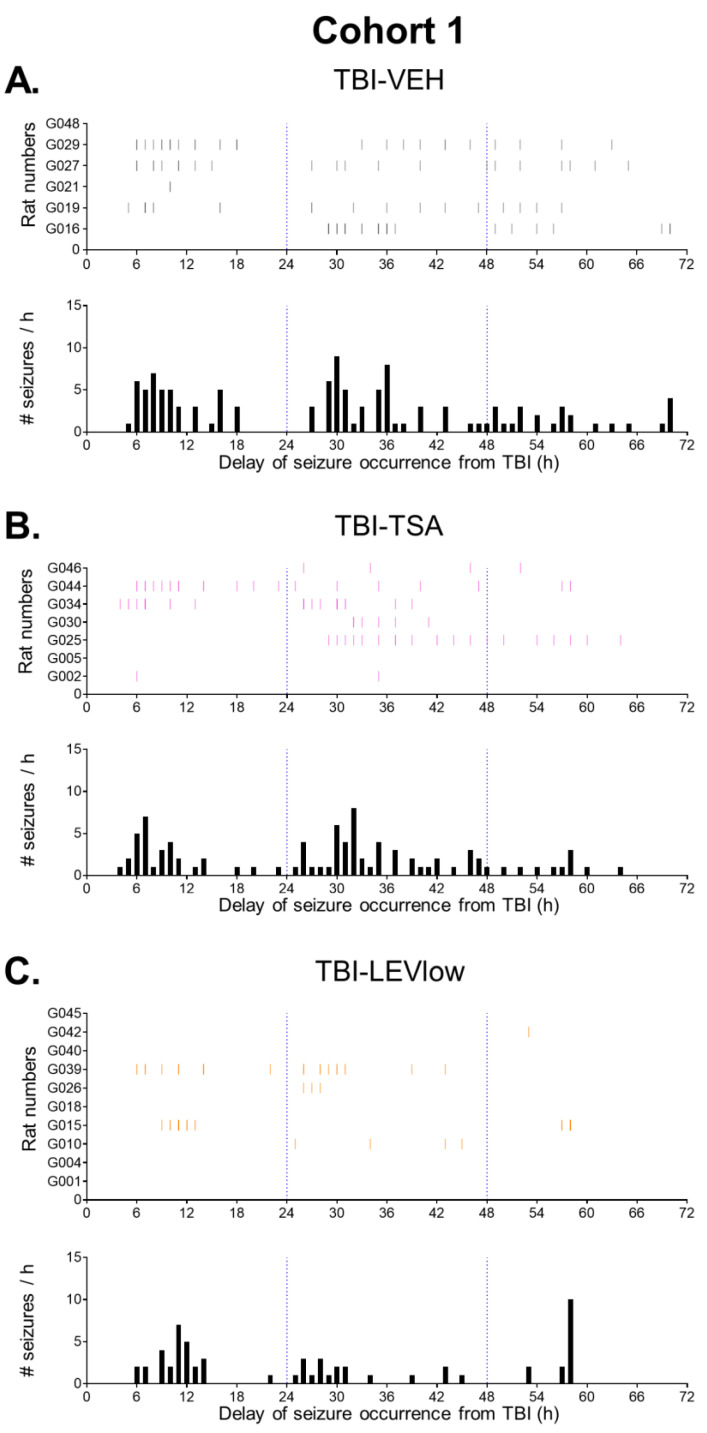
**Occurrence of seizures over the period of 0–72 h post-TBI—Cohort 1**. (**A**) TBI-VEH group. ***Upper panel***: A raster plot showing the occurrence of 119 seizures in 5 of 6 rats (*y*-axis). ***Lower panel***: A histogram showing the number of seizures per hour over the 72 h follow-up. (**B**) TBI-TSA group. ***Upper panel***: A raster plot showing the occurrence of 90 seizures in 6 of 7 rats (*y*-axis). ***Lower panel***: Histogram showing the number of seizures per hour over the 72 h follow-up. (**C**) TBI-LEV (lower dose, 54 mg/kg/d for 3 d) group. ***Upper panel***: Raster plot showing the occurrence of 60 seizures in 5 of 10 rats (*y*-axis). ***Lower panel***: Histogram showing the number of seizures per hour over the 72 h follow-up. Note that the increase in the number of seizures at 58 h was due to 1 rat, which had a cluster of seizures at that time point. Dashed blue lines divide the 72 h follow-up into 24 h epochs. **Abbreviations**: LEV, levetiracetam; TBI, traumatic brain injury; TSA, trichostatin A; VEH, vehicle; #, Number of.

**Figure 5:**
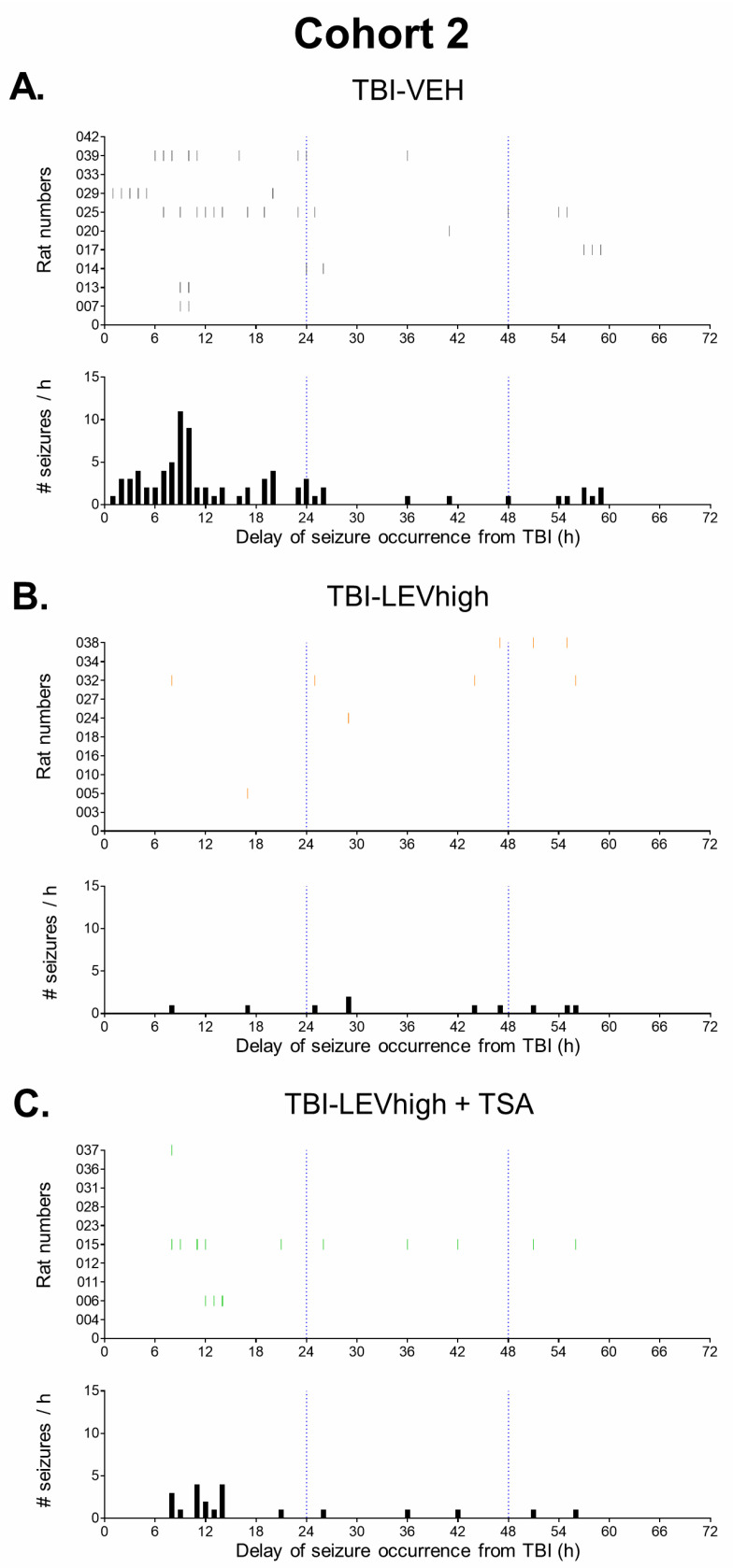
**Occurrence of seizures over the period of 0–72 h post-TBI—Cohort 2.** (**A**) TBI-VEH group. ***Upper panel***: A raster plot showing the occurrence of 79 seizures in 8 of 10 rats (*y*-axis). ***Lower panel:*** A histogram showing the number of seizures per hour over the 72 h follow-up. (**B**) TBI-LEV (higher dose, 150 mg/kg/d for 7 d) group. ***Upper panel***: A raster plot showing the occurrence of 10 seizures in 4 of 10 rats (*y*-axis). ***Lower panel:*** A histogram showing the number of seizures per hour over the 72 h follow-up. (**C**) TBI-LEV + TSA group. ***Upper panel***: A raster plot showing the occurrence of 21 seizures in 3 of 10 rats (*y*-axis). ***Lower panel***: A histogram showing the number of seizures per hour over the 72 h follow-up. Dashed blue lines divide the 72 h follow-up into 24 h epochs. **Abbreviations**: LEV, levetiracetam; TBI, traumatic brain injury; TSA, trichostatin A; VEH, vehicle; #, Number of.

As summarized in Supplementary Table S6 and Figure 4 and Figure 5, in the TBI-VEH and TBI-TSA groups, the seizure frequency decayed spontaneously over the 72 h follow-up. In the LEV-treated animals with a reduction in seizure number already during the 0–24 h period, the seizure numbers remained suppressed over the entire 72 h follow-up without any further decay.

##### Seizure Duration

Data are summarized in Supplementary Tables S7 and S8.

In the TBI-VEH group *(n* = 16), the average seizure duration was 52 ± 35 s (median 59 s, range 0–126 s). The Kruskal–Wallis test revealed differences in the average duration of electrographic seizures between groups (*p* < 0.01).

The average seizure duration was reduced in all LEV-treated animal groups compared with the TBI-VEH group: 23 ± 27 s (median 16 s, range 0–73 s, *p* < 0.05) in the TBI-LEVlow group (*n* = 10), 19 ± 27 s (median 0 s, range 0–66 s, *p* < 0.05) in the TBI-LEVhigh group (*n* = 10) and 12 ± 22 s (median 0 s, range 0–64 s, *p* < 0.01) in the TBI-LEVhigh + TSA group (*n* = 10). A higher LEV dose was not superior to a lower dose (*p* > 0.05). TSA monotherapy (*n* = 7) showed no effect on seizure duration (49 ± 31 s, median 56 s, range 0–87 s, *p* > 0.05 compared with the TBI-VEH group). Co-administration of TSA (*n* = 10) did not strengthen the LEV efficacy (*p* > 0.05).

Analysis of the evolution of the average duration of acute seizures in 0–24 h, 25–48 h, and 49–72 h epochs is summarized in Supplementary Table S7. Friedman’s two-way ANOVA test revealed that the average duration of electrographic seizures was comparable across all time points in the TBI-VEH, TBI-TSA, TBI-LEV, TBI-LEVhigh, and TBI-LEV high + TSA groups. When only the rats with seizures were included in the analysis, however, Cohen’s delta indicated a moderate-to-large treatment effect size on seizure duration in rats on LEV treatment (Supplementary Table S8).

##### Cumulative Seizure Duration

Data are summarized in Table 2.

In the TBI-VEH group (*n* = 16), the average cumulative seizure duration over the 72 h follow-up was 727 ± 688 s (median 537 s, range 0–1833 s). The Kruskal–Wallis test revealed differences in the cumulative duration of electrographic seizures between groups (*p* < 0.01).

The average cumulative seizure duration was reduced in LEVhigh-treated animal groups compared with the TBI-VEH group: 42 ± 64 s (median 0 s, range 0–186 s, *p* < 0.01) in the TBI-LEVhigh group (*n* = 10) and 109 ± 282 s (median 0 s, range 0–898 s, *p* < 0.01) in the TBI-LEVhigh + TSA group (*n* = 10). A higher LEV dose was not superior to a lower dose (*p* > 0.05) even though Cohen’s delta revealed a moderate treatment effect. TSA monotherapy (*n* = 7) showed no effect on seizure duration (898 ± 937 s, median 596 s, range 0–2344 s, *p* > 0.05) compared with the TBI-VEH group. Co-administration of TSA (*n* = 10) did not strengthen the LEV efficacy (*p* > 0.05).

Analysis of the evolution of the average cumulative duration of acute seizures in the 0–24 h, 25–48 h, and 49–72 h epochs is summarized in Table 2.

Friedman’s two-way ANOVA test revealed that the average duration of electrographic seizures was comparable across all time points in the ***TBI-VEH***, ***TBI-TSA***, ***TBI-LEV***, ***TBI-LEVhigh***, and ***TBI-LEV high + TSA*** groups. In the LEVhigh-treated animals who showed a reduction in the cumulative seizure duration already during the 0–24 h period, the cumulative seizure duration remained short over the next 48 to 72 h with no further decay.

##### Behavioral Severity of Electrographic Seizures

Data are summarized in Supplementary Table S9.

The Racine scale was used to assess the behavioral severity of the electrographic seizures in the rats [34]. Of a total of 409 electrographic seizures, a Racine score could be assigned to 93% (380/409) of the seizures. The remaining 6% (29/409 seizures) were scored as unknown as the behavioral symptoms could not be assigned to the Racine scale (Andrade AES abstract [35]). Note that 1% (4/409 seizures) of electrographic seizures could not be assigned to the Racine scale as there was no video recording during seizure occurrence due to technical problems.

In rats with acute and assigned seizures in the TBI-VEH group, the average Racine score was 0.20 ± 0.26 (median 0.00, range 0.00–0.63). The Kruskal–Wallis test revealed no differences between the treatment groups. Cohen’s delta, however, revealed a large treatment effect size in rats treated with a higher dose of LEV. The average Racine score in the 0–24 h, 25–48 h, and 49–72 h epochs showed no evolution over the 72 h follow-up (Supplementary Table S9).

## 3. Discussion

Our primary objective was to determine whether standard antiseizure medication combined with a tissue repair enhancer would better improve seizure control and structural outcome after TBI than antiseizure medication alone. To optimize the selection of the tissue repair enhancer, we first implemented and validated the in silico-to-in vitro systems biology approach for identifying neuroprotective drugs that could be repurposed. In the present study, we administered the antiseizure drug LEV as a monotherapy or in combination with the tissue repair enhancer TSA to rats with severe TBI induced by lateral FPI. We had four major findings. (1) In silico analysis predicted gene networks involved in neuroprotection, oxidative stress, and neuroinflammation as targets to alleviate secondary injury. (2) Of the five top compounds identified to regulate these networks thus selected for in vitro validation in neuronal–microglial co-cultures, three (TSA, calpain inhibitor i, and chlorpromazine) demonstrated promising in vitro therapeutic effects, whereas two (geldanamycin and tranylcypromine) did not. (3) TSA, a compound selected for in vivo validation, had a mild mitigating effect on the TBI-induced increase in plasma pNF-H levels at 72 h post-injury and on cortical lesion volume at D14 post-injury. (4) LEV showed a remarkable acute antiseizure effect on electrographically recorded early seizures that was not augmented by TSA.

### 3.1. LEV Showed a Remarkable Antiseizure Effect on Early Seizures after Severe TBI

Treatment of acute TBI includes the prophylactic use of antiseizure drugs to prevent TBI-related early (≤7 d) seizures [36,37,38]. Although prophylactic use of LEV has not shown superiority in its antiseizure efficacy for the treatment of human post-TBI seizures compared with other antiseizure drugs, its pharmacokinetic profile, ease of use, safety, and lack of interactions with other ongoing treatments promotes its use over other antiseizure medications in both civilian and combat situations [36,39,40,41]. Also, in rat models of induced seizures and SE, LEV demonstrates good antiseizure efficacy at the doses used in the present study, particularly the higher dose of 150 mg/kg [42,43,44]. In TBI models, the effect of LEV on early or late (>7 d) seizures has not been tested, which is not surprising as seizure monitoring requires continuous EEG recording starting right after the induction of TBI. Previous studies, however, demonstrated the beneficial effects of LEV on other post-TBI outcomes, including memory, somatomotor behavior, and histology in FPI and controlled cortical impact models [45]. LEV also affects plasma biomarkers of brain damage in FPI and penetrating ballistic-like injury models [45,46]. A recent ex vivo electrophysiologic slice study suggested that LEV administered within 1 h post-injury reduced epileptiform activity when assessed 2 weeks later in a controlled cortical impact model of TBI [47]. An additional benefit of LEV for experimental use is its water solubility, allowing for chronic administration using subcutaneous minipumps to maintain constant drug levels, which is critical for the treatment of TBI-induced SE, lasting for 3 to 4 days [20,48,49]. Pharmacokinetic analyses of LEV administered to normal or TBI rats showed that LEV reaches the serum Tmax in approximately 1 h and brain Tmax in approximately 2 h, and that TBI does not affect LEV pharmacokinetics [49,50].

As in our previous study [20], 81% of vehicle-treated animals developed early post-TBI electrographic seizures, SE, and associated electrographic patterns defined according to the International League Against Epilepsy and the International Federation of Clinical Neurophysiology guidelines [51,52]. In untreated rats, most of the seizures were non-convulsive, decayed spontaneously at the end of the 72 h follow-up, and did not associate with seizure-related mortality. Like in humans and induced seizures in rodents, LEV remarkably suppressed early electrographic seizures after lateral FPI in rats in a dose-dependent manner [42,43,44]. The antiseizure effect was clear already during 0–24 h post-injury, lasting for the entire 72 h analysis period. It should be noted that in untreated (or vehicle-treated) animals, the seizures typically start to occur at approximately 9 h (median) after the impact, and approximately 87% of the seizures occur during the first 48 h. Comparable data are reported in humans with TBI [16,19]. As LEV administration was started at 1 h (or 2 h in the lower dose study) post-impact, the drug concentrations in the brain were expected to have reached the Tmax by the time the first seizures appeared. In correlation, LEV treatment resulted in reduced seizure occurrence, seizure frequency, and cumulative seizure duration. Cohen’s delta suggested a moderate favorable effect on the duration of individual seizures, even at a lower LEV dose of 54 mg/kg/d.

Despite its remarkable dose-dependent antiseizure effect, LEV treatment resulted in no clear reduction of the cortical lesion area when assessed at 2 weeks or 6 months after TBI. Our data suggest an important difference between TBI-induced SE and conventional models of SE induced by chemoconvulsants or electrical stimulation. In conventional models, brain damage is largely generated by the SE activity, and consequently, can be alleviated by antiseizure medications if the SE is discontinued [53]. After TBI, however, cortical pathology starts even before the appearance of epileptiform activity. Within a few hours, TBI-related and SE-related secondary injuries progress in parallel, possibly augmenting each other. Suppressing SE only with LEV appeared to have little or no effect on the overall cortical pathology.

### 3.2. Systems Biology Analysis Revealed Trichostatin A as the Most Promising TBI-Related Pathology-Modifying Compound

The bioinformatic LINCS analysis, comparing the TBI and compound-induced transcriptomic changes indicated inflammatory response, cell death and survival, organismal injury, and free radical scavenging as the most important common regulated network functions. We anticipated that the compounds targeting these networks most efficiently would mitigate the TBI-induced neuropathology. Consequently, in vitro validation studies of the five top-scoring compounds, geldanamycin, tranylcypromine, calpain inhibitor, chlorpromazine, and TSA, were performed using a well-established neuron–microglia co-culture model that has proved useful for testing neuroprotective and anti-inflammatory compounds [54,55,56]. As response biomarkers for treatment efficacy, we used an MAP-2 assay for neurodegeneration, TNFα for neuroinflammation, and nitric oxide for oxidative stress.

Geldanamycin inhibits the function of Hsp90 and is used as an antitumor drug with antibiotic properties [57,58]. Unexpectedly, geldanamycin at 100 nM concentrations was cytotoxic to both cortical neurons and BV2-microglial cells. Previous data on its cytoprotective effects are controversial. Supko and colleagues [58] reported cytotoxic and cytostatic properties of geldanamycin in several human tumor cell lines. Xiao N et al., 1999 [59], however, demonstrated the neuroprotective effects of a 178 nM concentration of geldanamycin against glutamate-induced toxicity in a mouse hippocampal cell line, a higher concentration than that showing cytotoxicity in our in vitro experiments. Taken together, the therapeutic profile of geldanamycin appears to vary in different preparations. These data emphasize the need for in vitro validation of in silico-selected compounds before in vivo assessment.

Tranylcypromine, a monoamine oxidase inhibitor, is used as an antidepressant drug [60]. Although tranylcypromine failed to improve neuronal survival, it reduced nitrite levels at concentrations of 1 µM and 10 µM. Previously, Huang J and colleagues, 2017 [61] reported that 10 µM tranylcypromine was neurotoxic to human-induced pluripotent stem cell-derived brain organoids. Other studies, however, found that 1–10 µM tranylcypromine promotes neuroprotection in cortical neuronal cultures [62]. Differences in the in vitro outcome between studies may relate to the different preparations used.

Calpain inhibitor I, also known as N-acetyl-leucinyl-leucinyl-norleucinal [63], confers cytoprotection by inhibiting the protease activity of calpains and the production of TNFα [64]. In our study, calpain inhibitor I was neuroprotective at a 50 µM concentration and reduced nitrite and TNFα levels in a dose-dependent manner in the 1 µM to 50 µM dose range. Its anti-inflammatory effect was even greater than that of the positive control, IL10. Previous studies reported that 2.5–7.5 µM calpain inhibitor I had a necrotic effect on primary retinal neurons and increased lactate dehydrogenase levels after a 6 h treatment [65]. To date, however, the data reported suggest variable effects of calpain inhibitor I on cerebral neurons.

Chlorpromazine is a first-generation antipsychotic drug that blocks dopamine D2, histamine H1, and muscarinic M1 receptors [66,67]. Our in vitro experiments demonstrated that chlorpromazine improved neuronal viability and decreased nitrite and TNFα levels in a dose-dependent manner. The concentration range of 1–50 µM chlorpromazine that we used matches serum levels between 10–67 µM measured in psychotic patients [68]. In correlation with the present data, chlorpromazine at a 0.02 to 20 µM concentration was reported to reduce the release of inflammation mediators IL1β and IL2 in a mixed glial culture [69]. At concentrations of 1–1000 nM, chlorpromazine showed neuroprotective effects from *growth medium deprivation*-induced toxicity in hippocampal neuronal cultures [70]. In summary, our in vitro data support previous observations of the neuroprotective properties of chlorpromazine.

Trichostatin A is a highly potent histone deacetylase I and II inhibitor that promotes neuroprotection by upregulating neuroprotective genes and regulating the inflammatory response [71]. TSA is an investigational drug with antifungal and antibiotic properties [72]. Our in vitro assessment revealed that at a 50 nM concentration, TSA has both neuroprotective and antioxidant effects. Already at a 10- to 50 nM concentration, TSA also had anti-inflammatory effects. In addition, Agudelo M et al. (2012) [73] found that at a 50 nM concentration, TSA was neuroprotective against alcohol-induced oxidative stress in the SK-N-MC human neuronal cell line. Ryu H 2003 and colleagues [74] showed that 3 nM TSA protected rat primary cortical neurons from *glutamate analog homocysteate*-induced oxidative stress. Taken together, several lines of in vitro evidence provide support that TSA has mitigating effects on TBI-induced pathologies.

Overall, the in vitro data revealed the promising therapeutic potential of TSA, chlorpromazine, and calpain inhibitor in a neuronal–microglia cell culture model. The cytotoxicity of geldanamycin and the meager neuroprotective effects of tranylcypromine in the cell culture model system emphasizes the need for in vitro validation of in silico-discovered compounds as the LINCS score used for compound selection does not inform on the net effect of the compounds in living cells. To select a compound for in vivo validation in a TBI rat model, we next scored the performance of the drugs based on their ability to promote neuronal viability and to reduce nitrite and TNFα levels [28]. Although calpain inhibitor had the highest score, we selected TSA for further in vivo analysis as it had a higher neuronal viability score and a better microscopic neuronal viability profile compared with calpain inhibitor. Moreover, it promoted neuroprotection almost as well as the positive control 1400 W and exhibited an anti-inflammatory capacity that exceeded the effect of positive control IL10.

### 3.3. Trichostatin A Monotherapy Showed a Mild Neuroprotective Effect In Vivo But Had a Meager Treatment Effect In Vivo

TSA is the most potent histone deacetylase (HDAC) Class I and II inhibitor at nanomolar concentrations, resulting in the expression of neuroprotective genes [75,76]. Both Class I and II HDACs, particularly HDAC2, 3, 4, 5, and 11, are expressed in the rat cerebral cortex and hippocampus, which are the most common epileptogenic regions in animal models and in humans with post-traumatic epilepsy [76]. Three independent studies in two different injury models suggest increased acute upregulation of HDAC activity after TBI. Zhang et al. (2008) [77] showed a reduction in hippocampal acetyl-histone H3 immunostaining at 24 h in the lateral FPI model. Gao et al. (2006) [78] reported a reduction in hippocampal CA1 and CA3 H3 acetylation to 15% at 6 h and 24 h after CCI injury, which normalized by 72 h. Sagarkar et al. (2019) [79] reported a three- to four-fold increase in HDAC2 and HDAC3 mRNA levels after single and repeated central weight-drop-induced mild TBI and elevated nuclear and cytosolic HDAC activity after mild TBI. Consistent with experimental data, Srivastava et al. (2020) [80] reported the upregulation of hippocampal HDAC1, 2, 4, 5, 6, 10, and 11 mRNA in patients operated on for drug-refractory medial temporal lobe epilepsy with hippocampal sclerosis, suggesting chronic upregulation of HDACs in damaged brain with epileptiform activity (i.e., reduced acetylation).

The little evidence available suggests that both the kinetics of TSA interactions with HDAC and the elimination half-life of TSA in rat brain are fast, occurring within 24 h after an intraperitoneal injection of 0.5 mg/kg, the dose needed to achieve efficient HDAC inhibition [81,82,83]. Apparently more important than the actual half-life of TSA for the structural and functional outcome is the duration of the transcriptional regulation induced by TSA following its target engagement. Sagarkar et al. (2019) [79] showed that after a 3-d treatment with 2 mg/kg/d of TSA, the TBI-induced three-fold increase in HDAC activity was reduced back to control levels. TSA also increased the acetylated H3-K9 levels to 60% above the control levels (or to 300% of that in vehicle-treated TBI tissue with reduced acetylated H3-K9 levels). Most importantly, several in vivo studies revealed beneficial recovery-enhancing effects of TSA administration. For example, in previous favorable stroke intervention studies in rat, TSA was administered at a dose of 0.5 mg/kg (s.c.) immediately after a permanent middle cerebral artery occlusion followed by another injection 12 h later [82] or as a single dose of 0.05 mg/kg (i.p.) 20 min before middle cerebral artery occlusion [84]. In favorable TBI studies, TSA was administered at a dose of 2 mg/kg (i.p.) at 24 h intervals for 3 d starting at 28 d after repeated mild TBI induced with a weight drop [79]. In cancer studies with chronic tumor growth, TSA was administered to rats at a dose of 0.5–5 mg/kg (s.c.) daily for 4 weeks with no toxicity [85]. In both brain injury cancer studies, favorable therapeutic effects were typically achieved at a single dose of 0.5–2 mg/kg/day when administered for 1–3 d. Based on these data, we hypothesized that the neuroprotective, anti-inflammatory, and antioxidant effects of TSA to improve the post-TBI outcome will be reached over the three first treatment days with a 24 h administration interval of 1 mg/kg of TSA.

Consistent with the results of previous studies, our preliminary analysis with 1 mg/kg of TSA monotherapy administered for 3 d post-injury revealed no remarkable adverse events. The acceptable safety profile after severe TBI and mild favorable effects on pNF-H, a plasma marker of axonal brain injury, and on cortical lesion area at 2 weeks post-injury encouraged us to analyze its effects on functional outcome. In the present analysis, we were interested in the effect of TSA treatment on tissue recovery in the presence of epileptiform activity, which occurs in 70–80% of rats with severe lateral FPI and can worsen the structural and functional outcome [14,15,16,17,18,19,20].

First, we assessed whether TSA would affect the occurrence and/or severity of early seizures after TBI. A previous study demonstrated no antiseizure effects of 0.5 mg/kg or 5 mg/kg of TSA on pentylenetetrazol-induced seizures in rats [86]. Our data expand previous observations by showing that also unprovoked early post-TBI seizures were not affected by TSA monotherapy as the prevalence of seizure occurrence, the delay to the first post-impact seizure, seizure frequency, and cumulative seizure duration were comparable to that in vehicle-treated TBI controls. Next, we assessed whether TSA would improve the antiseizure efficacy of LEV possibly by neutralizing the ictogenic inflammatory signals in neuronal tissue via its anti-inflammatory effects and preserving the inhibitory networks via its neuroprotective effects. LEV-TSA combination therapy, however, showed no superiority compared with LEV monotherapy during the 72 h monitoring period. Also, the cortical lesion area was not affected by adding TSA to the treatment regimen with LEV. Disappointingly, the neuroprotective, anti-inflammatory, and antioxidant effects of TSA on cortical neuron–microglia co-cultures in vitro did not translate to in vivo functional or structural benefits.

### 3.4. Hurdles in Translation—Why Were Favorable In Vitro Neuroprotective Effects of TBI Not Found In Vivo?

The major disappointment of the present study was that the robust neuroprotective, antioxidant, and anti-inflammatory effects of TSA in an in vitro cell culture model did not translate to a reduced lesion area or improved seizure control in vivo. This may relate to several aspects in the experimental approaches used. First, the systems biology approach in compound selection focused mainly on three pathologic mechanisms: neuronal cell death, neuroinflammation, and oxidative stress. TBI results in a more complex pathophysiology with the interaction of several other disease mechanisms that must be considered [13,87,88].

Another limitation could relate to (a) differences in the pathophysiologic mechanisms of the mouse cell culture model compared with the rat TBI model and/or (b) differences between human cell line—derived data from the LINCS database compared to the TBI-sig which was derived from rat brain. Further, our cell culture consisted of two types of cells, cortical neuronal cells and BV2-microglia, which may not predict the complexity of the cell types involved in post-TBI secondary damage. Wang and colleagues (2012) [89] showed that the neuroprotective effect of 100 nM TSA observed in mouse neuronal cultures translated to a favorable outcome in a mouse model of cerebral ischemia (1 mg/kg). It remains to be explored whether expanding the repertoire of cell culture models will improve the translation of efficacy from in vitro to in vivo.

Cortical lesion area was used as an indicator of neuroprotection. Expanding indicators of neuroprotective efficacy to other brain areas and expanding the analysis to different cell types might increase the sensitivity of detecting the neuroprotective effects. Also, expansion of EEG analysis to other EEG patterns (e.g., delta activity, lateralized periodic discharges, epileptiform discharges, or continuous spike trains) could provide better prognostic biomarkers for a favorable outcome rather than analysis of seizures only.

## 4. Materials and Methods

### 4.1. Generation of TBI Gene Expression Signature in the Perilesional Cortex (TBI-Sig)

Two data sets that were sampled at different post-injury time points (32 h and 3 months) were used to generate a list of differentially expressed genes to define the TBI expression signature (TBI-sig) as previously described [27]. Briefly, the transcriptomes used included (a) microarray analysis of gene expression in the perilesional cortical tissue sampled at 32 h after lateral FPI [90], and (b) RNA sequencing analysis of gene expression in the perilesional cortical tissue sampled at 3 months after lateral FPI (GEO: series accession number GSE80174). Differentially expressing genes (fold-change |1.5| and false discovery rate < 0.05) at 32 h and at 3 months after TBI were identified (TBI-sig) to extend the potential therapeutic time window for targeted regulation of the specific gene with the treatment.

### 4.2. Selection of Drugs for In Vitro Validation

**Figure 6:**
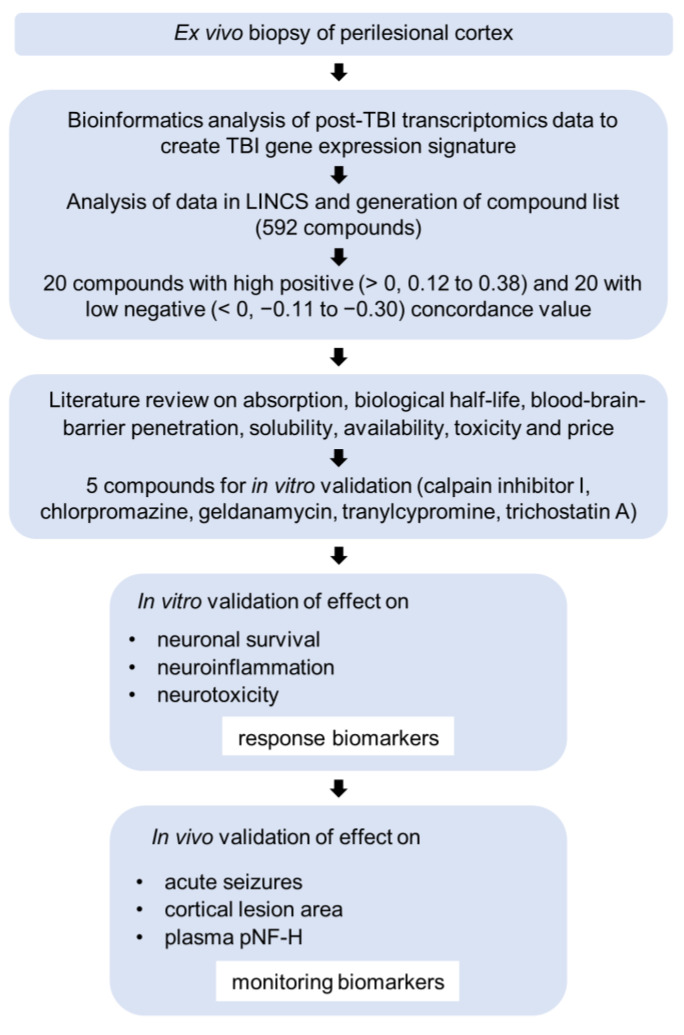
**Pipeline for selecting drugs for in vivo testing.** Perilesional transcriptomics data for bioinformatic analysis were collected at 32 h and 3 months after traumatic brain injury. LINCs analysis resulted in a list of 592 compound signatures derived from the LINCS database. Of these, 20 compounds with a high positive concordance and 20 compounds with a high negative concordance value were selected for further consideration. After a literature review, 5 compounds (calpain inhibitor I, chlorpromazine, geldanamycin, tranylcypromine, and trichostatin A) were chosen for in vitro validation using mouse primary cortical neuron BV2-microglial co-cultures. An immuno-based MAP-2 assay was used as a biomarker for neuronal survival, TNFα for neuroinflammation, and nitric oxide for oxidative stress/neurotoxicity. Finally, trichostatin A was selected for in vivo validation in the lateral fluid-percussion model of TBI. **Abbreviations:** LINCS, Library of Integrated Network-Based Cellular Signatures; MAP-2, microtubule-associated-protein 2; pNF-H, phosphorylated neurofilament heavy; TBI, traumatic brain injury; TNFα, tumor necrosis factor α.

Test compounds were selected from a list of compounds in the Library of Integrated Network-Based Cellular Signatures (LINCS) database [91] (http://www.ilincs.org/ilincs/, (accessed on 10 April 2018)). This list had concordance values ranging from −1 to +1 that predicted the correlation between the gene expression signature of a given compound in neuronal cultures (compound-sig) and the perilesional cortical TBI-sig. A positive concordance value suggested that both the compound and TBI-induced gene expression changes were in the same direction and a negative concordance value suggested the opposite direction [92]. Following a pipeline developed by Lipponen et al. (2019) [29] (Figure 6), a literature review was performed to search for the top 20 compounds with a high positive concordance value (0.100 to 0.387) and another 20 compounds with a high negative concordance value (−0.109 to −0.394) (Supplementary Table S1). We next collected information on solubility, blood–brain-barrier penetration, absorption, biologic half-life, toxicity, possible pharmacodynamic interactions, availability, and price of each of the top compounds. A compound was also considered if it was previously identified to have favorable disease-modifying effects in vivo for another condition without significant adverse events.

Next, the compound-sigs (i.e., list of genes regulated by a compound) of 20 compounds on the final list were retrieved from the LINCS database for further analysis using Ingenuity Pathway Analysis (IPA) software version 2.3 (Qiagen, Hilden, Germany). The compound-sig and perilesional TBI-sig were merged to produce a list of common differently expressed genes. The gene list was uploaded to IPA and a core analysis was performed to (a) create gene networks linked to genes on the list and (b) determine disease mechanisms associated with the gene networks. Analyses of the 32 h and 3-month data and the combined dataset were conducted separately (Supplementary Tables S2–S4).

Finally, based on all analyses, five compounds were selected for in vitro validation to determine the top candidate for in vivo assessment (Table 3).

### 4.3. In Vitro Validation of Compound Effects on Neuronal Viability, Oxidative Stress, and Neuroinflammation

**Figure 7:**
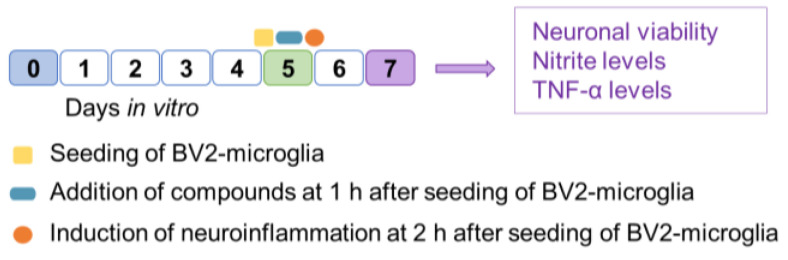
**Study design of in vitro validation of disease-modifying effects of the 5 compounds.** In vitro validation was performed using mouse primary cortical neuron BV2-microglial co-cultures. ***Day 0***: Culture of primary cortical neurons. **Day 5**: Immortalized BV2-microglia were seeded on top of the neurons. At 1 h later, test compounds were added to the medium. One hour later (i.e., 2 h after adding BV2-microglial cells), neuroinflammation was induced using LPS and IFNγ. Exposures were continued for 48 h. ***Day 7***: Medium was collected for assessment of nitrite and TNFα levels. The co-cultures were then fixed for assessment of neuronal viability by MAP-2 immunohistochemistry. **Abbreviations:** IFNγ, interferon γ; LPS, lipopolysaccharides; MAP-2, microtubule-associated-protein 2; TNFα, tumor necrosis factor α.

The study design of in vitro experiments is summarized in Figure 7.

#### 4.3.1. In Vitro Assessment of Neuroprotective, Antioxidant, and Anti-Inflammatory Effects of Compounds

##### Preparation of Mouse Primary Cortical Neurons and BV-2 Co-Culture

***Cortical neuronal culture***. Brains were dissected from JAXC57BL/6J mice on embryonic day 18 (E18) and the cerebral cortex was carefully stripped of the meninges. Cortical tissue was digested with 0.125% trypsin (#15090046, Gibco, Thermo Fisher Scientific, Waltham, MA, USA) in Dulbecco’s Modified Eagle Medium (#BE12-614F Lonza, Bale, Switzerland) for 20 min at 37 °C. The trypsin reaction was stopped using plating medium containing 10% fetal bovine serum (FBS, #10270-106, Gibco, Thermo Fisher Scientific), 100 U/mL penicillin, and 100 μg/mL streptomycin (#DE17-602E Lonza, Bale, Switzerland) in Dulbecco’s Modified Eagle Medium. The digested cortices were centrifuged at 1600× *g* for 5 min at room temperature (RT) and the pellet was resuspended in plating medium. The cell suspension was filtered using a 40 µM cell strainer (#542040, Greiner Bio-One, Kremsmuenster, Austria). The filtrate was centrifuged at 1200× *g* for 5 min at RT. The remaining pellet was resuspended in neuronal culture medium containing Neurobasal™ feeding medium (#12348017, Gibco, Thermo Fisher Scientific) supplemented with 2% B27 (#17504044, Gibco, Thermo Fisher Scientific), 2 mM L-glutamine (#17-605E, Lonza), 100 U/mL penicillin, and 100 μg/mL streptomycin. Neurobasal™ feeding medium was then added to obtain a single-cell suspension. Cortical neurons were counted using a hemocytometer and Neurobasal™ feeding medium was added to reach a final density of 40 × 10^4^ cells/mL. Then, 20 × 10^4^ cells in feeding medium were plated on 1x poly-D-lysine hydrobromide (#P6407, Sigma Aldrich, St Louis, MO, USA)-coated 48-well cell culture plates and cultured at 37 °C in a 5% CO_2_ humidified atmosphere.

***Co-cultures***. Cortical neurons were co-cultured with BV-2-microglial cells after maintaining the neurons in culture for 5 d. Briefly, 1 confluent 15 cm plate of BV2-microglial cells cultured in RPMI™-1640 medium (#R0883, Sigma Aldrich) supplemented with 0.011% 100 U/mL penicillin, 100 μg/mL streptomycin, 0.011% L-glutamine, and 0.09% FBS was used. To eliminate dead BV2 cells, RPMI medium was removed and the cells were washed once with phosphate-buffered saline (PBS). Then, BV2-microglial cells were gently scraped into fresh RPMI medium to create a cell suspension. The suspension was centrifuged at 800× *g* for 3 min at RT and the remaining pellet was resuspended in the neuronal culture medium. Then, neuronal culture medium was added to obtain a single-cell suspension. BV2-microglia were counted using a hemocytometer to obtain a density of 20 × 10^4^ cells/mL. Next, the neuronal culture medium was removed from each well and BV2 microglia cells were seeded at a density of 40 × 10^3^ cells/mL in neuronal culture medium on top of the neurons to obtain a cortical neuron-BV2 microglia co-culture [54,55,56]. The final BV2-microglia:cortical neuron ratio was 1:5.

##### In Vitro Assessment of Treatments Effects

***Test compounds***. At 1 h after adding the BV2-microglia cells, co-cultures were treated with different concentrations of trichostatin, calpain inhibitor, chlorpromazine, geldanamycin, and tranylcypromine. Different concentrations were used to assess the therapeutic range and the toxicity of the compounds.

***Trichostatin*** A (#T1952, Sigma Aldrich) was purchased as a ready-made 5 mM solution dissolved in 100% dimethyl sulfoxide (DMSO). It was diluted in supplemented neuronal culture medium to final concentrations of 0.1 µM, 1 µM, and 5 µM.

**Calpain *inhibitor*** I (#A6185, Sigma Aldrich) and **chlorpromazine** (#Y0000507, Sigma Aldrich) were dissolved in 100% DMSO (#D2650, Sigma Aldrich) to obtain a stock solution of 10 mM. Both drugs were diluted in supplemented neuronal culture medium to final concentrations of 100 µM, 1 mM, and 5 mM.

***Tranylcypromin****e* (#P8511, Sigma Aldrich) was dissolved in distilled Millipore water to obtain a stock solution of 5 mM, which was diluted in supplemented neuronal culture medium to final concentrations of 100 µM and 1 mM.

***Geldanamycin*** (#SML1278, Sigma Aldrich) was purchased as a ready-made solution of 1 mg/mL (1.78 mM) in 100% DMSO. It was diluted in supplemented neuronal culture medium to final concentrations of 0.1 µM, 1 µM, and 10 µM.

At 1 h after seeding the BV2-microglia on top of the cortical neurons, 3.5 µL of drug solution was added to quadruplicate wells. In the case of tranylcypromine, 7 µL of 5 mM tranylcypromine solution was added to wells to obtain a final concentration of 100 µM in the cell culture medium. The final drug concentrations in the cell culture medium were 1 nM, 10 nM, and 50 nM for trichostatin; 1 µM, 10 µM, and 50 µM for chlorpromazine and calpain inhibitor; 1 µM, 10 µM, and 100 µM for tranylcypromine; and 1 nM, 10 nM, and 100 nM for geldanamycin.

***Assay Controls***. Anti-inflammatory cytokine, interleukin 10 (IL10, 50 µg/mL, #500-M128, Peprotech, Rocky Hill, NJ, USA; final concentration of 50 ng/mL), and the neuroprotective inducible nitric oxide synthase (iNOS) inhibitor 1400 W hydrochloride (2 mM, #1415, Tocris, Bristol, UK; final concentration of 20 µM) were used as positive controls.

***Induction of neuroinflammation***. After a 1 h drug treatment, neuroinflammation was induced using 200 ng/mL of lipopolysaccharide (LPS, #L5543, Sigma Aldrich) and 20 ng/mL interferon γ (IFNγ, #i4777, Sigma Aldrich). At 48 h after inducing neuroinflammation, cell culture supernatants were collected and stored at −20 °C until further analysis of nitrite and tumor necrosis factor α (TNFα) levels. As negative controls, we used 8 control wells, 4 containing cortical neurons only and another 4 containing both cortical neurons and BV-2-microglia cells without exposure to LPS/IFNγ-induced neuroinflammation.

#### 4.3.2. In Vitro Outcome Measures

##### Neuronal Viability

Co-cultured cells were fixed with 4% paraformaldehyde (PFA, #28908, Thermo Fisher Scientific) in phosphate-buffered saline (PBS) for 20 min, and then washed twice with PBS. Fixed cells were stored in PBS; the plates were covered with parafilm and kept at 4 °C.

Viability of the cortical neurons was assessed by microtubule-associated-protein (MAP)-2 immunostaining described by [93]. Briefly, cells were incubated in 0.3% H_2_O_2_ in methanol for 10 min at RT for permeabilization and to block endogenous peroxidase activity. Non-specific staining was blocked by incubating the cells in 1% bovine serum albumin (#A9647, Sigma Aldrich) and 10% normal horse serum (#S-2000, Vector Laboratories, Burlingame, CA, USA) in PBS for 20 min. This was followed by overnight incubation in primary mouse anti-MAP-2 antibody (1:2000, M9942, Sigma Aldrich) in blocking serum at 4 °C. Biotinylated secondary horse anti-mouse antibody (1:2000, BA-2000, Vector Laboratories) in blocking serum was then added and the cells were incubated at RT for 1 h. For visualization of the reaction product, the wells were incubated for 1 h at RT with ExtrAvidin-HRP (1:2000, E2886, Sigma Aldrich) dissolved in blocking serum. The cells were washed 3 times for 10 min each between incubations. TMB peroxidase (3,3′,5,5′-tetramethylbenzidine, #SK-4400, Vector Laboratories) was prepared in a darkroom or a room with red light following the manufacturer’s instructions and added to the cells. After a 10 min incubation in TMB substrate, the substrate solution was transferred to a microtiter plate and absorbance was measured using a microtiter plate reader (Infinite M200, Tecan, Mennedorf, Zurich, Switzerland) at 650 nm and the Magellan program. Wells processed without primary antibody were used as background controls. This experiment was repeated twice and data from both experiment batches were combined (2 independent experiments, each with 3 replicates). The following equation was used for normalizing the results:

$$Percentage\;of\;neuronal\;viability=\frac{absorbance\;(drug\;treated\;wells-LPS/INF\gamma +wells)}{absorbance(1400\;W\;treated\;wells-LPS/INF\gamma +wells)}\;\times \;\mathrm{100}\%$$

##### Nitrite Assay

To measure the amount of nitric oxide secreted into the cell culture medium, a nitrite assay was performed using the Griess reagent kit (#G-7921, Thermo Fisher Scientific, Molecular Probes). The kit detects nitrite formed by the spontaneous oxidation of nitric oxide present in the cell culture medium. To carry out the nitrite assay, standards were prepared from a stock solution following the manufacturer’s instructions. Samples were diluted in deionized water at a ratio of 1:4. Samples and standards were incubated in Griess reagent for 30 min at RT. Absorbance was measured at a wavelength of 548 nm using a microplate reader (Infinite M200, Tecan) and the Magellan program. This experiment was repeated twice and data from both experiment batches were combined (2 independent experiments, each with 4 replicates). The following equation was used for normalizing the results:

$$Percentage\;of\;nitrite=\frac{absorbance\;(drug\;treated\;wells-1400\;W\;treated\;wells)}{absorbance(LPS/INF\gamma +wells-1400\;W\;treated\;wells)}\;\times \;\mathrm{100}\%$$

##### TNFα ELISA from Cell Culture Medium

The concentration of TNF-α in the cell culture medium was detected using a mouse TNFα enzyme-linked immunosorbent assay (ELISA) kit (#88-7324-22, Invitrogen, Thermo Fisher Scientific), following the recommendations and dilutions given by the manufacturer. Absorbance was measured at a wavelength of 450 nm using the Infinite M200 Tecan plate reader and the Magellan program. The ELISA assay was performed once (1 independent experiment with 4 replicates). The following equation was used for normalizing the results:

$$Percentage\;of\;TNF\alpha =\frac{absorbance\;\left(drug\;treated\;wells-IL10\;treated\;wells\right)}{absorbance\left(LPS/INFg+wells-IL10\;treated\;wells\right)}\;\times \;\mathrm{100}\%$$

### 4.4. Selection of Compounds for In Vivo Validation

After analyzing the data obtained from in vitro assays, a scoring system was used to select a drug to be tested in vivo (Table 1 and Supplementary Table S5). A score ranging from 0 to 1 was allocated to a drug according to its ability to promote neuronal viability and reduce inflammation (TNFα) and oxidative stress (NO) [29]. In addition, visualization of microscope images provided additional information for selecting a drug suitable for in vivo validation (data not shown).

### 4.5. In Vivo Validation: Effect on Acute Seizures, Cortical Lesion Area, and Plasma Phosphorylated Neurofilament Heavy Chain (pNF-H) Levels

**Figure 8:**
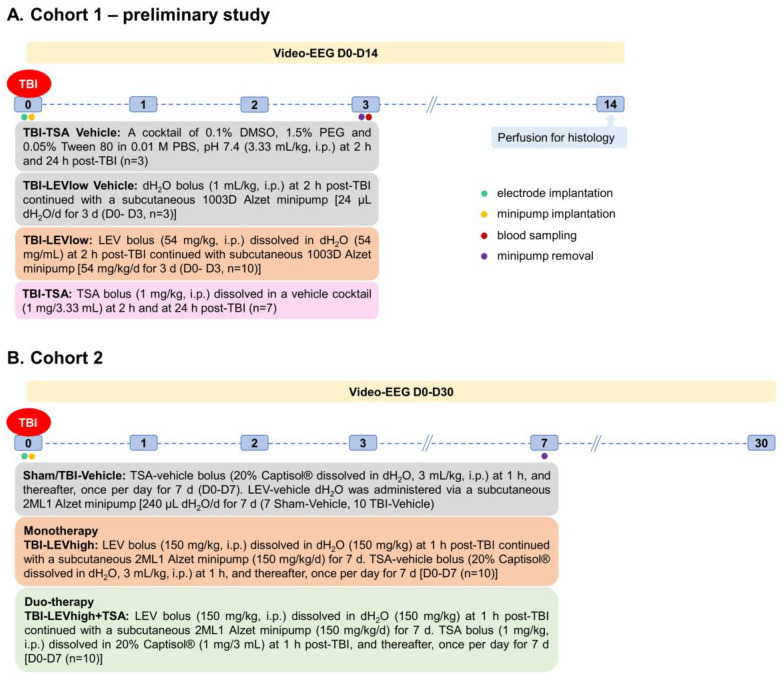
**Study design for in vivo validation**. Assessment of the disease-modifying effects of trichostatin A (TSA) on the structural and functional outcome after severe traumatic brain injury (TBI) was performed in cohort 1 using TSA as a monotherapy and in cohort 2 combining TSA with the antiseizure drug levetiracetam (LEV). Levetiracetam was chosen based on recent data indicating that severe TBI triggered by lateral fluid-percussion injury (FPI) induces epileptiform activity and seizures lasting for days, which can worsen the secondary damage and are not modified by TSA. Two separate cohorts were analyzed. (**A**) **Cohort 1**—a preliminary monotherapy study to assess the efficacy of LEV or TSA monotherapies on post-TBI outcome, mortality, and adverse events. (**B**) **Cohort 2**—a duo-therapy study. A higher dose of LEV was administered with or without TSA. Lateral FPI was performed on day (D) 0. In both cohorts, induction of injury and electrode implantation were performed in the same surgery session. Video-electroencephalography (video-EEG) was started immediately and continued till D14 (preliminary cohort) or D30. Treatments were initiated at 2 h (Cohort 1) or 1 h (Cohort 2) post-TBI and continued for 3 d (Cohort 1) or 7 d (Cohort 2) post-injury. In Cohort 1, we collected tail vein blood at 72 h post-injury to assess the injury and treatment effects on plasma levels of phosphorylated neurofilament heavy (pNF-H). In the end, rats were perfused for histology. **Abbreviations:** DMSO, dimethyl sulfoxide; dH_2_O, distilled water; PEG, polyethylene glycol; PBS, phosphate-buffered saline.

The randomization and study design are summarized in Figure 1 and Figure 8.

#### 4.5.1. Animals

Sixty-eight adult male Sprague Dawley rats (Envigo Laboratories, Horst, Limburg, Netherlands or, Loughborough, Leicestershire, UK, 10–12 weeks old and 350 ± 50 g at the time of sham-operation or TBI) were randomized into the different treatment groups. Rats were housed in individual plexiglass cages (42 cm × 26 cm × 18 cm, L × W × H) in a controlled environment (temperature 22 ± 2 °C; humidity 55 ± 15%, light–dark cycle from 07:00 to 19:00) with free access to water and pellet food (Teklad 2016S, Envigo).

All animal experiments were approved by The Animal Ethics Committee of the Provincial Government of Southern Finland and performed in accordance with the guidelines of the European Community Council Directives 2010/63/EU.

#### 4.5.2. Induction of Lateral Fluid-Percussion TBI

Severe TBI was induced with lateral FPI as previously described [94,95]. Briefly, the rat was placed into an anesthesia induction chamber and isoflurane anesthesia (Attane vet, #26675-46-7, Parola, Finland) was induced at 5% (room air as carrier gas; Somnosuite, # SS6069B, Kent Scientific, Torrington, CT, USA). The anesthetized rat was mounted in a stereotaxic frame (David Kopf Instruments, Tujunga, CA, USA), and then a probe was inserted into the rectum to continuously assess core temperature and a heating pad was placed below the abdomen. The temperature of the heating pad was regulated based on the animal core temperature (max 38 °C). Isoflurane was delivered via a nose cone mounted on the stereotaxic frame and maintained at 2 ± 0.2% throughout the surgery. The scalp incision site was shaved and cleaned using sterile 0.9% NaCl before subcutaneous injection with 0.5% lidocaine (3 mg/kg, 5 mg/mL subcutaneously (s.c.); Orion Pharma, Espoo, Finland). Approximately 3–5 min later, a midline incision was made and the surface of the skull was cleaned. A craniotomy, 5 mm in diameter (center coordinate: anteroposterior (AP) −4.5 mm from bregma; mediolateral [ML] 2.5 mm) was made over the left cortex using a handheld trephine (# 18004-50; Fine Science Tools GmbH, Heidelberg, Baden-Württemberg, Germany) with the dura left intact. A plastic female Luer-lock connector was inserted into the craniotomy vertical to the skull surface and its edges were sealed with tissue-adhesive glue (3M VetbondTM, 3M Deutschland GmbH, Neuss, North Rhine-Westphalia, Germany). Autopolymerizing dental acrylate (Selectaplus powder #10009210; Selectaplus liquid CN #D10009102; DeguDent, Hanau, Hesse, Germany) was spread around the Luer-lock and the connector set-up was secured to the skull with an ipsilateral frontal screw. To induce TBI, the Luer-lock was filled with 0.9% NaCl and the rat was connected to a straight-tipped fluid-percussion device (Model FP 302, AmScien Instruments, Richmond, VA, USA). The pressure was adjusted to produce severe injury with an anticipated mortality rate of 20–30% within the first 48 h. The mean impact pressure was 2.8 ± 0.13 atm (range: 2.1–3.0 atm). After injury, the rat was disconnected from the fluid-percussion device and placed on a heating pad; a rectal temperature probe was used for monitoring recovery.

The occurrence of post-impact seizure-like behavior and its duration, duration of the post-impact apnea, and time to return of the righting reflex were recorded. Sham-operated controls underwent all surgical procedures except exposure to FPI. All surgical procedures including TBI induction and electrode implantations were performed by the same person to reduce the impact of experimenter-induced variability on the experimental outcome.

#### 4.5.3. Electrode Implantation

In cohort 1, 22 of the 26 rats were implanted with both epidural and intracerebral electrodes. In cohort 2, all 37 rats included in the follow-up were implanted with epidural electrodes only. Electrodes were implanted immediately after the lateral FPI using coordinates based on a rat brain atlas [96]. In brief, after the return of the righting reflex, the rat was re-anesthetized with 2 ± 0.2% isoflurane and placed in a stereotaxic frame.

**Figure 9:**
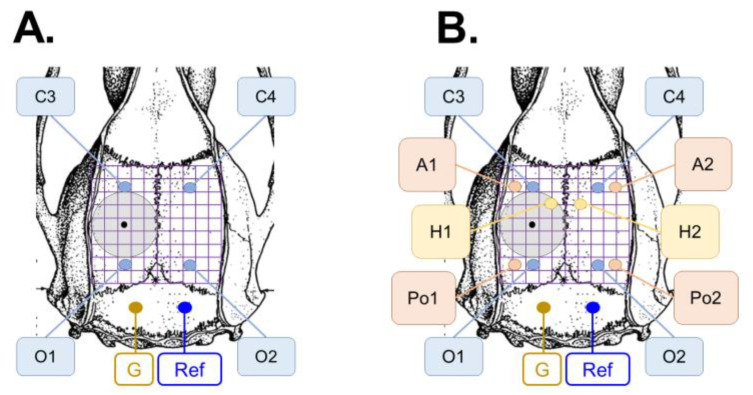
**Electrode montage.** (**A**) ***Epidural electrodes***. Four of 23 rats in Cohort 1 and all 37 animals in Cohort 2 that were included in the follow-up had 4 epidural electrodes only (C3, C4, O1, and O2). (**B**) ***Epidural and intracerebral electrodes***. In Cohort 1, 19/23 rats in the final analysis cohort also had 2 anterior intracortical (A1 (left)/A2 (right)), 2 posterior intracortical (Po1/Po2), and 2 intrahippocampal (H1/H2) electrodes, in addition to the 4 epidural electrodes. Epidural ground (G) and reference (R) electrodes were implanted above the cerebellum. Grey circle indicates the craniectomy, the black dot indicates the center of the craniectomy. Grid dimensions 1 mm × 1 mm.

***Epidural electrodes***. Four recording epidural stainless-steel screw electrodes (EM12/20/SPC; Bilaney Consultants GmbH, Dusseldorf, Germany) were implanted into the skull: 2 ipsilaterally (left frontal cortex C3: AP −1.7, ML 2.5; parieto-occipital cortex O1: AP −7. 6, ML 2.0) and 2 contralaterally (right frontal cortex C4: AP 1.7, ML 2.5; parieto-occipital cortex O2: AP −7.6, ML 2.0) (Figure 9A).

***Intracerebral electrodes***. Altogether, 6 intracerebral tungsten monopolar recording electrodes (EM12/3-2TW/SPC; thickness 50 µm, Bilaney Consultants GmbH, Dusseldorf, Germany) were implanted (Figure 9B). On the ipsilateral side (left), 3 were implanted with the following coordinates: anterior cortex A1 (AP −1.7, ML 4.0 and DV 2.2), posterior cortex Po1 (AP −7.6, ML 4.0, DV 1.8), hippocampus H1 (AP: −3.0, ML 1.4, DV 3.6). On the contralateral side (right), 3 electrodes were implanted with the following coordinates: anterior cortex A2 (AP −1.7, ML 4.0 and DV 2.2), posterior cortex Po2 (AP −7. 6, ML 4.0, DV 1.8), hippocampus H2 (AP −3.0, ML 1.40, DV 3.6).

In addition, 1 epidural screw electrode serving as ground was placed ipsilaterally posterior to lambda, and another serving as a reference electrode was placed contralaterally. One (epidural electrodes only) or two 6-pin connectors were secured to the skull using autopolymerizing dental acrylate (see above). Electrodes were attached to a multi-pin connector (MS12P, PlasticsOne Inc., Roanoke, VA, USA) with monopolar referential montage.

#### 4.5.4. Preparation and Implantation of Subcutaneous Alzet Minipumps

A day before surgery, Alzet osmotic minipumps (Model 1003D or 2ML1, DURECT Corporation, Cupertino, CA, USA) were filled with vehicle (Sterile Millipore^®^ water, Burlington, MA, USA) or levetiracetam (see Section 4.5.10) and incubated overnight at 37 °C in 0.9% NaCl to ensure immediate drug delivery after implantation. The next day, immediately after electrode surgery, minipumps were implanted subcutaneously into the interscapular space. During pump implantation, anesthesia was maintained using 2 ± 0.2% isoflurane inhalation.

#### 4.5.5. Post-Impact Monitoring and Care

For analgesia, rats were administered buprenorphine (0.05 mg/kg, s.c.; Orion Pharma, Finland) immediately after electrode implantation. Powdered pellet food and 0.9% NaCl (10 mL, s.c.) were given to the rats 1–2 times a day for 3 d post-injury or until the rats could eat solid pellet food and drink water independently. The well-being of the rats was monitored daily by assessing for signs of pain or discomfort, postoperative bleeding, appearance of the coat, and bowel and gastrointestinal function.

#### 4.5.6. Video-Electroencephalography (Video-EEG) Monitoring

For video-EEG monitoring, the electrode headset attached to the rat skull was connected to a 6-pin swivel commutator (SL12C, PlasticsOne Inc.) via a flexible shielded cable (363-363, PlasticsOne Inc.), allowing the rat to move freely during the EEG recordings. The commutator was connected to an amplifier with a flexible shielded cable (363/2-441/12 (PlasticsOne Inc.)). High-fidelity electrical brain activity was monitored using a 320-channel Digital Lynx 16SX amplifier (Neuralynx, Bozeman, MT, USA) with a 10 kHz sampling rate. The amplifier had an analog bandwidth between DC to 80 kHz.

The amplifier had 80 independent analog references, allowing for a configuration of independent references for each animal. Data from each channel were converted individually into 24 bits. Each animal was video-monitored with a single high-resolution camera (Basler acA1300-75gm GigE, Basler, Ahrensburg, Germany) configured to record 30 frames per second (maximum 75) with a resolution of 1.3 megapixels and compressed using H.264. At night, cameras recorded under cage-specific WFL-II/LED15W infrared illumination (24 V, 150 mA). The EEG and video were synchronized using the precision time protocol IEEE-1588. The entire system generated approximately 1.5 TB of data every 24 h. For data storage, the video-EEG system was connected to a network-attached storage server (Synology RS4017xs+) with 200 TB of storage in a RAID6 configuration for redundancy and checksum for data integrity. As the capacity of the Neuralynx system was 30 rats per time, a few animals were monitored using the Nicolette system (see Nissinen et al., 2017 [97]).

#### 4.5.7. Video-EEG Analysis

***Electrographic seizures***. EEG files were converted using NCS2SMRX (version 0.38). Manual seizure detection was then performed by browsing the files using the Spike 2 software (version 10).

The following outcome measures were assessed: (1) latency to the first seizure, (2) number of electrographic seizures between 0–72 h post-impact, (3) duration of electrographic seizures between 0–72 h post-impact, and (4) cumulative seizure duration over the 72 h follow-up.

***Behavioral severity of electrographic seizures***. Videos were synchronized to the EEG files. The rat behavior associated with electrographic seizures was scored using a Racine scale [34].

#### 4.5.8. Randomization of Animals into Treatment Groups

Randomization is summarized in Figure 1.

***Cohort 1***. The objective was to assess the feasibility of the planned study design and obtain preliminary data on the antiseizure effect of Levetiracetam (LEV) and the neuroprotective effect of Trichostatin A (TSA) as monotherapies on the post-TBI outcome. Altogether, 26 rats were injured with lateral FPI. Three rats were excluded (1 with disconnection of Luer-lock during impact, 1 with broken dura, 1 with prolonged post-impact recovery and low oxygen saturation). Consequently, 23 rats were randomized into the vehicle (TBI-VEH, *n* = 6), LEV (TBI-LEV, *n* = 10), or TSA (TBI-TSA, *n* = 7) treatment groups.

***Cohort 2***. The objective was to determine (a) the effect of a higher LEV dose and longer treatment duration on the outcome, and (b) whether the LEV-TSA duo-therapy would be more effective than LEV monotherapy on the post-TBI outcome. A total of 42 rats were randomized into the sham-operated (*n* = 8) or lateral FPI-induced TBI (*n* = 34) groups. In the sham-operated group, 1 had a broken dura and was excluded. In the TBI group, 4 rats died. Consequently, 7 rats were included in the sham-VEH group and 30 into the TBI treatment groups (TBI-VEH, *n* = 10; TBI-LEV, *n* = 10; TBI-LEV + TSA, *n* = 10).

#### 4.5.9. Drug Preparation

***Trichostatin A—Cohort 1.*** *TSA* (# 58880-19-6, Cayman Chemical, MI, USA) was dissolved in a vehicle cocktail containing 0.1% DMSO (#D2650, Sigma Aldrich), 1.5% polyethylene glycol (PEG, #202398, Sigma Aldrich), and 0.05% Tween 80 (#P1754, Sigma Aldrich) in PBS to obtain a solution of 0.3 mg/mL (pH 7.4). To improve the solubility of TSA, it was sonicated (V011 m-Range, Model m3) for 30 min at 30 °C to produce a milky solution. Then, aliquots were stored at −20 °C until thawed on the day of administration.

***Trichostatin A—Cohort 2.*** *TSA* (# 58880-19-6, MedChemExpress, Bergkallavägen, Sweden) was dissolved in Captisol^®^ (sodium sulfobutyl ether beta-cyclodextrin # 182410-00-0, Acros Organics, Geel, Belgium) to obtain a solution of 0.3 mg/mL. To improve the solubility of TSA, it was sonicated as described above. The TSA was prepared fresh every day.

***Levetiracetam. (A) Bolus.*** LEV (#102767-28-2, SynInnova, Edmonton, AB, Canada) was dissolved in sterile Millipore^®^ water (54 mg/mL or 150 mg/mL).

**(B) *Minipump. Cohort 1***—LEV (54 mg/kg/d) was dissolved in dH_2_O and administered via an Alzet subcutaneous minipump model 1003D (DURECT Corporation; 1.3 mg/24 µL/24 h). ***Cohort 2***—LEV (150 mg/kg/d) was dissolved in dH_2_O and administered via Alzet subcutaneous minipump model 2ML1 (DURECT Corporation; 3.6 mg/240 µL/24 h).

#### 4.5.10. Drug Administration

##### Cohort 1

***TBI*-TSA *VEH (n = 3).*** The vehicle cocktail (0.1% DMSO, 1.5% PEG and 0.05% Tween in PBS) used to dissolve TSA was administered at 2 h and 24 h post-TBI (3.33 mL/kg, intraperitoneally (i.p.)).

***TBI-LEV VEH (n = 3). (A) Bolus.*** dH_2_O (1 mL/kg, i.p.) was administered as a bolus at 2 h post-TBI. ***(B) Minipump.*** Then, the vehicle treatment was continued via Alzet mini-pumps (1 µL/h, s.c.) on D0–D3.

***TBI-LEV (n = 10). (A) Bolus***. LEV (#102767-28-2, SynInnova, Edmonton, Canada) was dissolved in dH_2_O and administered as a bolus (54 mg/kg, i.p.) at 2 h post-TBI to prevent acute post-TBI seizures and SE. ***(B) Minipump***. To ensure stable plasma levels, LEV administration was continued via subcutaneous Alzet minipumps (54 mg/kg/d) on D0–D3. Justification of the dose was based on previous in vivo studies in mice and rats [45,98].

***TBI-TSA (n = 7).*** TSA (1 mg/kg) was administered intraperitoneally at 2 h and 24 h post-TBI. Justification of the dose was based on previous in vivo studies in mice and rats [82,89].

##### Cohort 2

***Sham-VEH. (A) TSA vehicle***. A bolus of 20% Captisol^®^ in dH_2_O (3 mL/kg, i.p.) was administered at 1 h post-operation, and then once per day on D1–D7. ***(B) LEV vehicle.*** dH_2_O administered via Alzet mini-pumps (10 µL/h, s.c.) on D0–D7.

***TBI-VEH. (A) TSA vehicle***. A bolus of 20% Captisol^®^ in dH_2_O (3 mL/kg, i.p.) was administered at 1 h post-TBI, and then once per day on D1–D7. ***(B) LEV vehicle.*** dH_2_O administered via Alzet mini-pumps (10 µL/h, s.c.) on D0–D7.

***TBI-LEV.* *(A) Bolus.*** LEV (#102767-28-2, SynInnova, Edmonton, AB, Canada) dissolved in dH_2_O and administered as a bolus (150 mg/kg, i.p.) at 1 h post-TBI. ***(B) Minipump.*** LEV administration was continued via subcutaneous Alzet minipumps (150 mg/kg/d) on D0–D7.

***TBI-LEV + TSA. (A) Bolus***. LEV (#102767-28-2, SynInnova, Edmonton, AB, Canada) dissolved in dH_2_O and administered as a bolus (150 mg/kg, i.p.) at 1 h post-TBI. TSA (1 mg/kg) in 20% Captisol^®^ was administered intraperitoneally at 1 h post-TBI, and then, every 24 h on D1-D7. ***(B) Minipump***. LEV administration was continued via subcutaneous Alzet minipumps (150 mg/kg/d) on D0–D7.

#### 4.5.11. Blood Sampling and Plasma Preparation

In cohort 1, blood was sampled at 72 h post-TBI to assess the treatment effect on pNF-H levels (Figure 8). Blood sampling from the tail vein and plasma sample preparation were performed as described earlier [99,100]. Briefly, rats were anesthetized by isoflurane inhalation (5% induction, and 1–2% maintenance). A 23 G butterfly needle (#367247, BD Vacutainer, Franklin Lakes, NJ, USA) was used to draw 1 mL of blood from the lateral tail vein into 2 BD Microtainer K_2_ EDTA-tubes (#365975, di-potassium ethylenediaminetetraacetic acid, BD Biosciences; 500 µL/tube). Each tube was manually inverted twice to mix whole blood with EDTA and then placed on ice until centrifugation. To obtain plasma, tubes were centrifuged at 1300× *g* for 10 min at 4 °C (5417R Eppendorf, Hamburg, Germany) within 1 h after blood sampling. The plasma hemolysis coefficient was measured at 414 nm using a spectrophotometer (NanoDrop^®^ ND1000 Spectrophotometer, ND-1000 v3.8.1, Thermo Fisher Scientific, Waltham, MA, USA). Plasma aliquots of 50 µL were carefully collected and pipetted into 0.5 mL Protein LoBind tubes (#022431064, Eppendorf, Hamburg, Germany), flash-frozen on dry ice, and stored at −70 °C.

#### 4.5.12. Quantification of Plasma pNF-H

Plasma levels of pNF-H were detected using a human pNF-H ELISA assay kit (#RD191138300R, BioVendor R&D^®^, Brno, Czech Republic), following the manufacturer’s recommendations. Samples were diluted in dilution buffer at a ratio of 1:6. Samples and standards were incubated in substrate buffer for 20 min at RT. Then, absorbance was measured at 630 nm (reference wavelength) and at 450 nm using a microplate reader (Wallac Victor^2^, Perkin Elmer, CA, USA). Absorbance readings at 630 nm were subtracted from those at 450 nm.

#### 4.5.13. Histology

Details of the histology [101] and preparation of cortical unfolded maps [32] were previously described and are only briefly summarized here.

***Perfusion***. On D14 (cohort 1) or D180 (cohort 2), deeply anesthetized rats were intracardially perfused with 0.9% NaCl followed by 4% PFA in 0.1 M sodium phosphate buffer (PB). The brain was removed from the skull and fixed in 4% PFA for 4 h, cryoprotected in 20% glycerol in 0.02 M potassium phosphate-buffered saline (pH 7.4) for 24–36 h, frozen in dry ice, and stored in −70 °C for further processing. Frozen coronal sections of the brain were cut (30-µm thick, 1-in-5 series) using a sliding microtome. The first series of sections was stored in 10% formalin at room temperature and used for thionin staining. Other series of sections were collected into tissue collection solution (30% ethylene glycol, 25% glycerol in 0.05 M PB) and stored at −20 °C until processed.

***Nissl staining***. The first series of sections was stained with thionin, cleared in xylene, and cover-slipped using Depex^®^ (BDH Chemical, Poole, UK) as a mounting medium.

***Preparation of cortical unfolded maps.*** To assess the cortical lesion area and the damage to different cytoarchitectonic cortical areas after TBI as well as the treatment effect, thionin-stained sections were digitized (40×, Hamamatsu Photonics, Hamamatsu, Japan; NanoZoomer-XR, NDP.scan 3.2). Unfolded cortical maps were then prepared from the digitized histologic sections as described in detail by [32] and by applying in-house software from https://unfoldedmap.org (accessed on 14 November 2022) adapted to the rat brain [102].

#### 4.5.14. Statistical Analysis

Statistical analyses were performed using R software (version 3.5.1) (http//www.R-project.org/ (accessed on 1–15 November 2019)) or SPSS for Windows (v. 27.0). The effect of a given compound on neuronal viability, oxidative stress, and neuroinflammation in vitro was analyzed using a linear regression model (normalizing batch effect) in R with the lm function. The treatment effect on the occurrence of seizures in different groups was tested using Fisher’s exact test. Treatment and time effects on seizure duration, cumulative seizure duration, and the Racine score were analyzed using repeated-measures analysis of variance (ANOVA), followed by post hoc Bonferroni correction for multiple tests. Within each treatment group, the evolution of changes was assessed using Friedman’s two-way ANOVA, followed by post hoc Bonferroni correction for multiple tests. Intra-timepoint comparisons between the animal groups were tested with the nonparametric Kruskal–Wallis test, followed by post hoc analysis with the Mann–Whitney U test (i.e., seizure duration, cumulative seizure duration, and Racine score). Cohen’s delta was assessed as an indicator of effect size. Pearson’s test was used to analyze the correlation between the pNF-H levels and cortical lesion area. Data are shown as the mean ± standard deviation of the mean (unless otherwise specified). A *p*-value < 0.05 was considered significant.

## 5. Conclusions

***Towards Rational Polytherapy in the Treatment of SE after TBI***. We present data from the first preclinical intervention study on the efficacy of anti-seizure drugs with or without tissue recovery enhancer on post-TBI acute seizures. As observed clinically, prophylactic LEV administration effectively suppressed seizures after lateral FPI-induced TBI, validating the use of the severe TBI model in anti-seizure drug testing. The antiseizure effect, however, was not accompanied by a reduced cortical lesion area when assessed 6 months later. These data demonstrate that in structural SE etiologies such as TBI, antiseizure treatment is not sufficient to improve structural outcomes and co-administration of a tissue-pathology-repairing compound is required. Disappointingly, the systems biology-identified tissue-repairing compound, TSA, with its robust favorable in vitro effects on tissue biomarkers of neuroprotection, neuroinflammation, and oxidative stress, did not augment the early antiseizure effect or chronic structural outcome of LEV in vivo. It remains to be explored whether using multiple in vitro culture models, expanding the repertoire of in vitro monitoring biomarkers, testing the treatment in a milder TBI model, or expanding the number of clinically relevant outcome measures (e.g., memory, sleep, evolution of post-traumatic epilepsy) will provide better guidance for compound selection for translational studies.

## Figures and Tables

**Table 1:** Scoring of in vitro-validated drugs based on their effect on neuronal viability, nitrite production, and TNFα levels. Scoring criteria are summarized in Supplementary Table S5. Based on scoring, trichostatin A was forwarded to in vivo validation. Please note that calpain inhibitor had the highest score, but it was not selected for in vivo validation because of its in vitro toxicity at higher concentrations detected in microscopic analysis of exposed cell cultures.

Compound	Concentration	Neuronal Viability	Nitrite	TNFα	Total Score
Trichostatin A	50 nM	0.75	0.75	1	2.5
7,8-dihydroxychlorpromazine	50 µM	0.45	0.30	1	1.75
Calpain inhibitor	50 µM	0.60	1	1	2.60
(+)-Tranylcypromine	1 µM	0.15	0.15	N/A	0.30
Geldanamycin	10 nM	0.15	0.15	N/A	0.30

**Abbreviations:** TNFα, tumor necrosis factor α.

**Table 2:** Average cumulative duration of seizures (s) per rat, in different treatment groups between 0–72 h after traumatic brain injury (TBI). Cumulative seizure duration is also shown in 24 h epochs (0–24 h, 25–48 h, 49–72 h).

Treatment Group	All Seizures (K-W 0.005)	Time after TBI (h)			Intragroup Statistics (Friedman’s Two-Way ANOVA)
		T1 = 0–24 h (K-W 0.070)	T2 = 25–48 h (K-W 0.009)	T3 = 49–72 h (K-W 0.383)	
TBI-Veh (16)	727 ± 688 (198) [537, 0–1833]	438 ± 510 (110) [208, 0–1406]	169 ± 290 (57) [10, 0–953]	120 ± 176 (31) [0, 0–475]	ns
TBI-TSA (7)	898 ± 937 (90) [596, 0–2344] (Cohen’s d −0.222)	372 ± 632 (31) [0, 0–1391] (Cohen’s d 0.120)	414 ± 330 (49) [596, 0–734] (Cohen’s d −0.813)	112 ± 175 (10) [0, 0–419] (Cohen’s d 0.053)	ns
TBI-LEVlow (10)	358 ± 715 (60) [41, 0–2111] (Cohen’s d 0.528)	201 ± 444 (28) [0, 0–1287] (Cohen’s d, 0.487)	67 ± 146 (18) # [0, 0–462] (Cohen’s d, 0.414)	95 ± 258 (14) [0, 0–824] (Cohen’s d, 0.123)	ns
TBI-LEVhigh (10)	42 ± 64 (10) **, # [0, 0–186] (Cohen’s d 1.256) C d to LEVlow 0.623	10 ± 22 (2) * [0, 0–66] (Cohen’s d 1.060) C d to LEVlow 0.607	14 ± 23 (5) # [0, 0–58] (Cohen’s d 0.675) C d to LEVlow 0.511	18 ± 49 (3) [0, 0–154] (Cohen’s d 0.719) C d to LEVlow 0.414	ns
TBI-LEVhigh + TSA (10)	109 ± 282 (21) **, # [0, 0–898] (Cohen’s d 1.083)	62 ± 137 (16) [0, 0–424] (Cohen’s d 0.912)	29 ± 92 (3) ## [0, 0–291] (Cohen’s d 0.593)	18 ± 58 (2) [0, 0–183] (Cohen’s d 0.713)	ns

Data are shown as the mean ± standard deviation of the mean. Animal numbers are shown in parentheses (in column “Treatment Group”). Median and range are shown in brackets. **Abbreviations**: C d to LEVlow, Cohen’s delta to TBI-LEVhigh treatment group vs. TBI-LEVlow treatment group; h, hour; K-W, Kruskal–Wallis test; LEVlow, levetiracetam 54 mg/kg/d; LEVhigh, levetiracetam 150 mg/kg/d; ns, not significant; TBI, traumatic brain injury; TSA, trichostatin A; Veh, vehicle. **Statistical significance**: Differences between treatment groups at each time interval were tested using the Kruskal–Wallis test. Differences between the groups were analysed using Mann–Whitney U test: *, *p* < 0.05; ** *p* < 0.01 compared with the TBI-Veh group; #, *p* < 0.05; ##, *p* < 0.01, compared with the TBI-TSA group. Time, treatment group, and time x treatment group effects were tested using a general linear model with Bonferroni correction. There were differences in average cumulative seizure duration between the treatment groups (*p* < 0.05). Differences across the time intervals (0–24 h, 25–48 h, 49–72 h) within each treatment group were tested using a related-samples Friedman’s 2-way ANOVA with Bonferroni correction for multiple testing (right column). In each cell, the Cohen’s delta between the TBI treatment group vs. the TBI vehicle group (in parentheses) showed moderate (≥0.50) or large (≥0.80) effect sizes.

**Table 3:** LINCS analysis. Concordance value of the five compounds selected for in vitro analysis at 32 h and 3 months post-TBI and the cell line.

Compound	32 h		3 Months	
	Concordance Value	Cell Line	Concordance Value	Cell Line
Trichostatin A	0.331	Neu	0.373	Neu
Geldanamycin	0.281	Neu-KCL	0.375	Neu-KCL
Calpain inhibitor	0.268	Neu-KCL	0.367	Neu-KCL
7,8-dihydroxychlorpromazine	0.221	Neu-KCL	0.226	Neu-KCL
(+)-Tranylcypromine	−0.206	Neu-KCL	−0.251	Neu-KCL

***Abbreviations:*** LINCS, Library of Integrated Network-Based Cellular Signatures; Neu, neurons; Neu-KCL, potassium chloride-treated neurons.

## Data Availability

The data presented in this study are available on request from the corresponding author.

## Supplementary Materials

The supporting information can be downloaded at https://www.mdpi.com/article/10.3390/ijms241814049/s1.
