# Supplementary material for: Treatment of Status Epilepticus after Traumatic Brain Injury Using an Antiseizure Drug Combined with a Tissue Recovery Enhancer Revealed by Systems Biology
Source: Int J Mol Sci. 2023 Sep 13;24(18):14049. doi: 10.3390/ijms241814049 (PMC10531083; doi:10.3390/ijms241814049)
Supplement: Supplementary file 1 [file ijms-24-14049-s001.zip › ijms-2575599-SI/Supplementary Figures/Supplementary Figure - S1A - D - Figure and Legend.pdf]

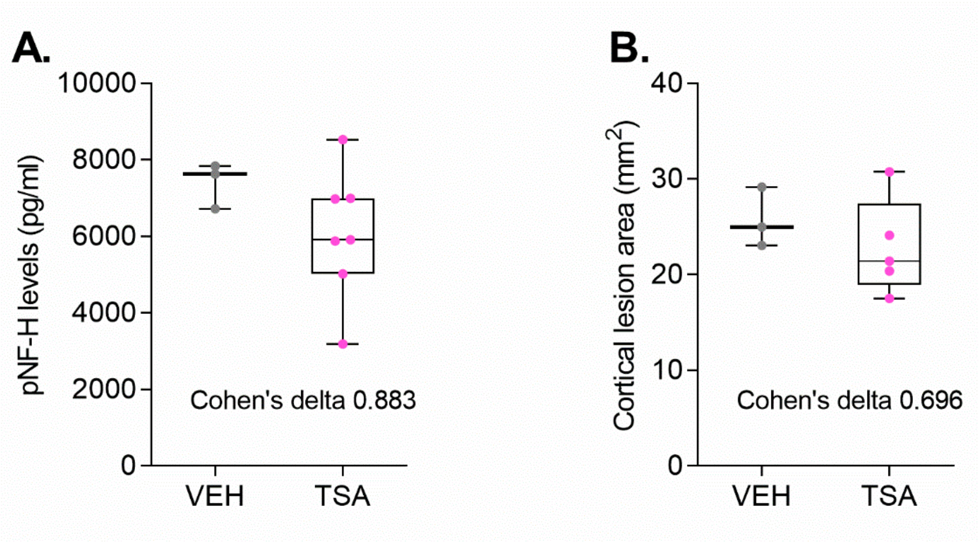
A.
10000
8000
6000
4000
2000
0
pNF-H levels (pg/ml)
Cohen's delta 0.883
VEH
TSA
B.
40
30
20
10
0
Cortical lesion area (mm²)
Cohen's delta 0.696
VEH
TSA

C. Rat G039

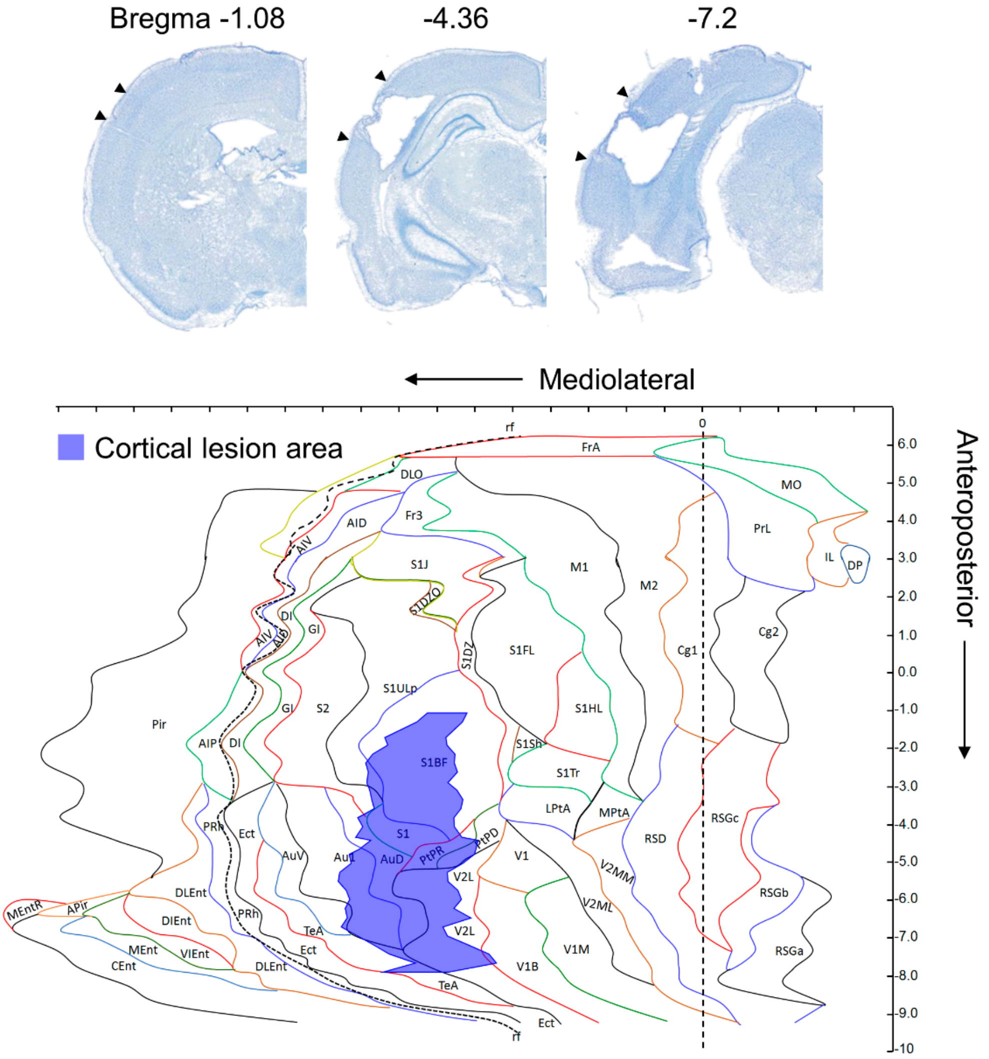
Bregma -1.08
-4.36
-7.2
Mediolateral
Anteroposterior
Cortical lesion area
rf
FrA
MO
PrL
IL
DP
DLO
Fr3
AID
AIV
S1J
M1
M2
Cg1
Cg2
S1DZO
S1DZ
S1FL
S1HL
S1ULp
S2
GI
DI
AIP
Pir
S1BF
S1Sh
S1Tr
LPtA
MPtA
RSD
RSGc
RSGb
RSGa
S1
PtPR
PtPD
AuD
AuV
Au1
Ect
PRh
V2L
V1
V1B
V1M
V2MM
V2ML
TeA
DLEnt
DIEnt
MEnt
VIEnt
CEnt
APir
MEntR
0
6.0
5.0
4.0
3.0
2.0
1.0
0.0
-1.0
-2.0
-3.0
-4.0
-5.0
-6.0
-7.0
-8.0
-9.0
-10

D. Rat G040

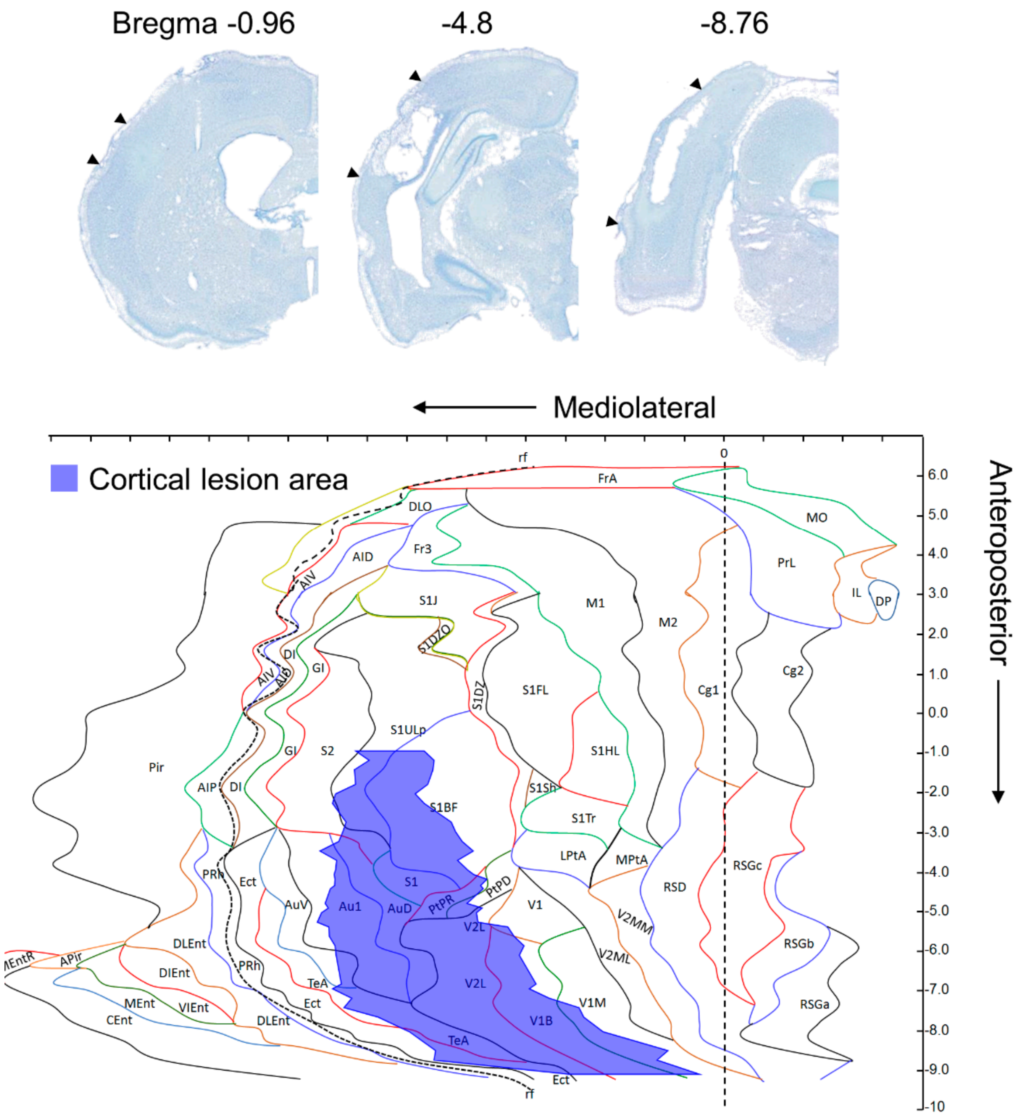

Supplementary Figure S1. ***Effect of TSA*** **on** ***plasma pNF-H and cortical lesion area.*** **(A)** In vehicle-treated rats with traumatic brain injury (TBI), pNF-H levels (y-axis) varied between 6 721 to 7 835 pg/ml. Even though the average plasma pNF-H levels did not differ between the TBI-VEH and TBI-TSA groups ($p > 0.05$, Kruskal-Wallis), Cohen's delta (0.885) indicated a moderate favorable effect size of TSA treatment. **(B)** The cortical lesion area (y-axis) in the TBI-VEH group ranged from 23.1 to 29.2 mm^2. Although the average cortical lesion area did not differ between the TBI-VEH and TBI-TSA groups ($p > 0.05$, Kruskal-Wallis), Cohen's delta (0.696) indicated a mild favorable effect size of TSA treatment. (**C,D)** Examples of cortical lesion areas of 2 rats (G039 and G040). Rat G039 in panel C (TBI-LEV, lower dose) had a cortical lesion area of 15.8 mm^2 and plasma pNF-H level of 448 pg/ml. Rat G040 (TBI-LEV, lower dose) in panel D with a cortical lesion area of 29.9 mm^2 and plasma pNF-H level of 8 721 pg/ml. The upper panels show coronal thionin-stained sections from 3 rostro-caudal levels (left is most rostral and right, right is most caudal; numbers refer to distance from bregma according to the rat brain atlas of Paxinos [2007]). The lower panel shows the unfolded cortical map of the cortical lesion (violet area) prepared according to Ndode-Ekane et al. (2017). ***Abbreviations***: LEV, levetiracetam; pNF-H, phosphorylated neurofilament heavy; TSA, trichostatin A; VEH, vehicle.
