# Supplementary material for: Treatment of Status Epilepticus after Traumatic Brain Injury Using an Antiseizure Drug Combined with a Tissue Recovery Enhancer Revealed by Systems Biology
Source: Int J Mol Sci. 2023 Sep 13;24(18):14049. doi: 10.3390/ijms241814049 (PMC10531083; doi:10.3390/ijms241814049)
Supplement: Supplementary file 1 [file ijms-24-14049-s001.zip › ijms-2575599-SI/Supplementary Tables S1- S9/Supplementary Table S1 - LINCS score of top 40 compounds .pdf]

**Supplementary Table S1.** Top scoring 20 compounds with the highest (positive) or lowest (negative) concordance score based on LINCS analysis. Compounds highlighted with a green shading were selected for *in vitro* validation based on criteria specified in the text.

| Compound | 32 h | | 3 months | |
|---|---|---|---|---|
| | **Concordance Value** | **Cell line** | **Concordance value** | **Cell line** |
| Tanespimycin | 0.387 | NEU | 0.180 | NEU |
| Tacedinaline | 0.366 | NEU | 0.328 | NEU |
| Trichostatin A | 0.331 | NEU | 0.373 | NEU |
| Phenoxybenzamine | 0.289 | NPC | 0.228 | NPC |
| Geldanamycin | 0.281 | NEU.KCL | 0.375 | NEU.KCL |
| Calpain inhibitor I | 0.268 | NEU.KCL | 0.367 | NEU.KCL |
| BML-284 | 0.264 | NEU.KCL | 0.195 | NEU.KCL |
| 7,8-dihydroxychlorpromazine | 0.221 | NEU.KCL | 0.226 | NEU.KCL |
| No-asa | 0.204 | NPC | 0.248 | NPC |
| Indirubin-3'-monoxime | 0.193 | NPC | 0.153 | NPC |
| Dihydrocapsaicin | 0.187 | NPC | 0.231 | NPC |
| Fenretinide | 0.181 | NEU.KCL | 0.332 | NEU.KCL |
| MG-132 | 0.177 | NEU | 0.297 | NEU |
| Tamoxifen | 0.174 | NPC | 0.124 | NPC |
| Vorinostat | 0.172 | NEU | 0.189 | NEU |
| 15d-pgj2 | 0.152 | NEU.KCL | 0.140 | NEU.KCL |
| Oxamflatin | 0.141 | NEU.KCL | 0.234 | NEU.KCL |
| isonicotinamide | 0.127 | NEU.KCL | 0.174 | NEU.KCL |
| 5-nitroso-8-cinnolinol | 0.126 | NEU.KCL | 0.232 | NEU.KCL |
| BNTX | 0.100 | NEU.KCL | 0.383 | NEU.KCL |
| Calcitriol | -0.131 | NEU | -0.193 | NEU |
| Gemfibrozil | -0.145 | NEU | -0.187 | NEU |
| Tetradecylthioacetic acid | -0.158 | NPC | -0.227 | NPC |
| Tretinoin | -0.199 | NEU.KCL | -0.252 | NEU.KCL |
| (+)-tranylcypromine | -0.206 | NEU.KCL | -0.251 | NEU.KCL |
| schembl5962596 | -0.212 | NPC | -0.264 | NPC |
| CPNQ | -0.212 | NPC | -0.248 | NPC |
| Phenylbutanoic acid | -0.218 | NEU.KCL | -0.188 | NEU.KCL |
| Anisomycin | -0.252 | NEU | -0.109 | NEU |
| FK 866 | -0.256 | NEU.KCL | -0.224 | NEU.KCL |
| Rosiglitazone | -0.258 | NEU.KCL | -0.229 | NEU.KCL |
| QS-11 | -0.275 | NEU.KCL | -0.214 | NEU.KCL |
| Chembl27449 | -0.286 | NPC | -0.297 | NPC |
| Glucosamine | -0.302 | NEU.KCL | -0.263 | NEU.KCL |
| Progesterone | -0.306 | NEU.KCL | -0.249 | NEU.KCL |
| 2-chloro-10-ethyl-10h-phenothiazine | -0.312 | NEU.KCL | -0.203 | NEU.KCL |
| Merodantoin | -0.313 | NEU.KCL | -0.206 | NEU.KCL |
| Dactolisib | -0.340 | NEU.KCL | -0.192 | NEU.KCL |
| Spermidine | -0.359 | NEU.KCL | -0.203 | NEU.KCL |
| Cycloheximide | -0.394 | NEU | -0.191 | NEU |

***Abbreviations:*** LINCS, Library of Integrated Network-Based Cellular Signatures; Neu, neurons; Neu-KCL, potassium chloride-treated neurons.
