# Supplementary material for: Treatment of Status Epilepticus after Traumatic Brain Injury Using an Antiseizure Drug Combined with a Tissue Recovery Enhancer Revealed by Systems Biology
Source: Int J Mol Sci. 2023 Sep 13;24(18):14049. doi: 10.3390/ijms241814049 (PMC10531083; doi:10.3390/ijms241814049)
Supplement: Supplementary file 1 [file ijms-24-14049-s001.zip › ijms-2575599-SI/Supplementary Tables S1- S9/Supplementary Table S2 - IPA TBI sig at 32 h and compound sig .pdf]

Supplementary Table S2. Ingenuity Pathway Analysis (IPA) of network functions of overlapping genes between the compound-signature and the TBI-signature at 32 h after TBI. The score is a measure of the number of eligible molecules in a network. The greater the number of network-eligible molecules, the higher the score. The score is inversely proportional to the p-value. Blue text indicates mechanisms investigated in vitro.

Compound	Total number of gene networks	Top 3 networks	Score
Calpain inhibitor I	6	Cellular development, Cellular growth and proliferation, Cell cycle	33
		**Cell death and survival**, Connective tissue development and function, Connective tissue disorders	33
		Cellular movement, Cardiac enlargement, Cardiovascular disease	20
Chlorpromazine	3	Cell cycle, connective tissue development and function, Hematological system development and function	20
		Cell cycle, Hematological system development and function, **Inflammatory response**	17
		Cancer, Amino acid metabolism, Small molecule biochemistry	6
Geldanamycin	7	Cancer, Endocrine system disorders, **Organismal injury** and abnormalities	34
		**Cell death and survival, Organismal injury** and abnormalities, Cardiovascular disease	25
		**Cell death and survival, Organismal injury** and abnormalities, **Inflammatory response**	21
Tranylcypromine	6	Cancer**, Organismal injury** and abnormalities, Reproductive system disease	33
		Cancer, **Organismal injury** and abnormalities, Renal and urological disease	31
		Cancer, **Organismal injury** and abnormalities, Reproductive system disease	18
Trichostatin A	3	**Cell death and survival**, Cancer, **Organismal injury** and abnormalities	28
		Cell morphology, Cellular function and maintenance, Organ morphology	26
		**Humoral immune response**, Protein synthesis, Hematological system development and function	23

Networks are ranked according to an IPA score. ***Abbreviations***: IPA, Ingenuity Pathway Analysis; TBI, traumatic brain injury
