# Supplementary material for: Treatment of Status Epilepticus after Traumatic Brain Injury Using an Antiseizure Drug Combined with a Tissue Recovery Enhancer Revealed by Systems Biology
Source: Int J Mol Sci. 2023 Sep 13;24(18):14049. doi: 10.3390/ijms241814049 (PMC10531083; doi:10.3390/ijms241814049)
Supplement: Supplementary file 1 [file ijms-24-14049-s001.zip › ijms-2575599-SI/Supplementary Tables S1- S9/Supplementary Table S3 - IPA TBI sig at 3 months and compound sig .pdf]

Supplementary Table S3. Ingenuity Pathway Analysis (IPA) of network functions of overlapping genes between the compound-signature and the TBI-signature at 3 months after TBI. The score is a measure of the number of eligible molecules in a network. The greater the number of network-eligible molecules, the higher the score. The score is inversely proportional to the p-value. Blue text indicates mechanisms investigated *in vitro*.

Compound	Total number of gene networks	Top 3 networks	Score
Calpain inhibitor I	3	**Inflammatory response**, Cancer, Hematological disease	38
		Cellular movement, Cancer, Cellular development	26
		Cellular development, Organismal development, Hematological system development and function	15
Chlorpromazine	2	Cell morphology, Cellular function and maintenance, Cellular movement	37
		Gene expression, Cell cycle, Respiratory system development and function	9
Geldanamycin	8	Gene expression, **Cell death and survival, Organismal injury** and abnormalities	24
		Cancer, **Organismal injury** and abnormalities, Respiratory disease	21
		**Cell death and survival, Organismal injury and abnormalities,** Cellular development	21
Tranylcypromine	6	Cell morphology, Embryonic development, Hematological system development and function	24
		Glomerular injury, **Inflammatory disease, Inflammatory response**	22
		Cardiovascular system development and function, Cell cycle skeletal and muscular system development and function	16
Trichostatin A	4	Cancer, Cellular development, Cellular growth and proliferation	31
		Cancer, **Organismal injury** and abnormalities, Reproductive system	25
		Cancer, Neurological disease, **Organismal injury** and abnormalities	17

Networks are ranked according to an IPA score. ***Abbreviations***: IPA, Ingenuity Pathway Analysis; TBI, traumatic brain injury.
