# Supplementary material for: Treatment of Status Epilepticus after Traumatic Brain Injury Using an Antiseizure Drug Combined with a Tissue Recovery Enhancer Revealed by Systems Biology
Source: Int J Mol Sci. 2023 Sep 13;24(18):14049. doi: 10.3390/ijms241814049 (PMC10531083; doi:10.3390/ijms241814049)
Supplement: Supplementary file 1 [file ijms-24-14049-s001.zip › ijms-2575599-SI/Supplementary Tables S1- S9/Supplementary Table S4 - IPA TBI sig at 32h and 3 months combined and compound sig.pdf]

Supplementary Table S4. Ingenuity Pathway Analysis (IPA) of network functions of overlapping genes between the compound-signature and the TBI-signature at 32 h and 3 months after TBI. The score is a measure of the number of eligible molecules in a network. The greater the number of network-eligible molecules, the higher the score. The score is inversely proportional to the p-value. Blue text indicates mechanisms investigated *in vitro*.

Compound	Total number of gene networks	Top 3 networks	Score
Calpain inhibitor I	1	**Free radical scavenging**, Molecular transport, Cell to cell signalling and interaction	15
Chlorpromazine	2	Cell signalling, Molecular transport, Vitamin and mineral metabolism	27
		Connective tissue disorders, Developmental disorder, Hereditary disorder	2
Geldanamycin	5	Cellular growth and proliferation, Connective tissue development and function, Tissue development	22
		Cancer, **Organismal injury** and abnormalities, Cellular development	22
		**Cell death and survival, Organismal injury** and abnormalities, Cell cycle	19
Tranylcypromine	4	Cancer, Endocrine system disorders, **Organismal injury** and abnormalities	27
		Lymphoid tissue structure and development, Tissue morphology, Infectious diseases	16
		Cellular movement, Cardiovascular disease, Cardiovascular system development and function	8
Trichostatin A	2	Cancer, Cellular development, Cellular growth and proliferation	39
		Connective tissue development and function, Skeletal and muscular system development and function, Tissue development	11

Networks are ranked according to an IPA score. ***Abbreviations***: IPA, Ingenuity Pathway Analysis; TBI, traumatic brain injury.
