# Supplementary material for: Treatment of Status Epilepticus after Traumatic Brain Injury Using an Antiseizure Drug Combined with a Tissue Recovery Enhancer Revealed by Systems Biology
Source: Int J Mol Sci. 2023 Sep 13;24(18):14049. doi: 10.3390/ijms241814049 (PMC10531083; doi:10.3390/ijms241814049)
Supplement: Supplementary file 1 [file ijms-24-14049-s001.zip › ijms-2575599-SI/Supplementary Tables S1- S9/Supplementary Table S5 - Scoring criteria based on in vitro outcome.pdf]

Supplementary Table S5: Scoring criteria based on ***in vitro*** outcome measures (neuronal viability, nitrite levels, TNF-α levels).

***In vitro* outcome**	**Score**							
	**0**	**0.15**	**0.3**	**0.45**	**0.6**	**0.75**	**0.9**	**1**
% neuronal viability	≤ 0	>15	>30	>45	>60	>75	>90	≥100
% nitrite levels	≥100	<90	<75	<60	<45	<30	<15	≤0
% TNFα levels	≥100	<90	<75	<60	<45	<30	<15	≤0

***Abbreviations*:** TNFα, tumor necrosis factor α.
