# Supplementary material for: Treatment of Status Epilepticus after Traumatic Brain Injury Using an Antiseizure Drug Combined with a Tissue Recovery Enhancer Revealed by Systems Biology
Source: Int J Mol Sci. 2023 Sep 13;24(18):14049. doi: 10.3390/ijms241814049 (PMC10531083; doi:10.3390/ijms241814049)
Supplement: Supplementary file 1 [file ijms-24-14049-s001.zip › ijms-2575599-SI/Supplementary Tables S1- S9/Supplementary Table S6 - Average number of seizures between 0-72 h post TBI .pdf]

Supplementary Table S6. Average number of seizures in different treatment groups between 0–72 h after traumatic brain injury (TBI). Number of seizures is also shown in 24-h epochs (0–24 h, 25–48 h, 49–72 h).

Treatment Group	All seizures<br>(K-W 0.007)	Time after TBI (h)			Intragroup statistics<br>(*Friedman's two-way ANOVA*)
		**T1=0–24 h**<br>(K-W 0.061)	**T2=25–48 h**<br>(K-W 0.010)	**T3= 49–72 h**<br>(K-W 0.360)	
TBI-Veh (13/16)	12.4 ± 12.7 (198)<br>[8.0, 0.0–40.0]	6.9 ± 7.7 (110)<br>[2.0, 0.0–20.0]	3.6 ± 7.8 (57)<br>[0.5, 0.0–31.0]	1.9 ± 3.0 (31)<br>[0.0, 0.0–9.0]	Friedman's two-way analysis p=0.014<br>T3-T1 adj. p=0.051
TBI-TSA (6/7)	12.9 ± 11.4 (90)<br>[10.0, 0.0–27.0]<br>(*Cohen's d* -0.039)	4.4 ± 7.4 (31)<br>[0.0, 0.0–18.0]<br>(*Cohen's d* 0.321)	7.0 ± 5.8 (49)<br>[6.0, 0.0–14.0]<br>(*Cohen's d* -0.474)	1.4 ± 2.3 (10)<br>[0.0, 0.0–6.0]<br>(*Cohen's d* 0.183)	Friedman's two-way analysis p=0.048<br>T3-T2 adj. p=0.098
TBI-LEVlow (5/10)	6.0 ± 10.5 (60)<br>[1.0, 0.0–29.0]<br>(*Cohen's d* 0.535)	2.8 ± 6.1 (28)<br>[0.0, 0.0–17.0]<br>(*Cohen's d* 0.571)	1.8 ± 3.6 (18) $^{\#}$<br>[0.0, 0.0–11.0]<br>(*Cohen's d* 0.271)	1.4 ± 3.8 (14)<br>[0.0, 0.0–12.0]<br>(*Cohen's d* 0.164)	ns
TBI-LEVhigh (4/10)	1.0 ± 1.5 (10) $^{**,\#}$<br>[0.0, 0.0–4.0]<br>*(Cohen's d 1.131)*<br>*C d to LEVlow 0.666*	0.2 ± 0.4 (2) *<br>[0.0, 0.0–1.0]<br>*(Cohen's d 1.095)*<br>*C d to LEVlow 0.604*	0.5 ± 0.9 (5) $^{\#}$<br>[0.0, 0.0–2.0]<br>*(Cohen's d 0.498)*<br>*C d to LEVlow 0.503*	0.3 ± 0.7 (3)<br>[0.0, 0.0–2.0]<br>*(Cohen's d 0.690)*<br>*C d to LEVlow 0.405*	ns
TBI-LEVhigh +TSA (3/10)	2.1 ± 4.6 (21) $^{**,\#}$<br>[0.0, 0.0–14.0]<br>*(Cohen's d 0.987)*	1.6 ± 3.2 (16)<br>[0.0, 0.0–9.0]<br>*(Cohen's d 0.825)*	0.3 ± 0.9 (3) $^{\#\#}$<br>[0.0, 0.0–3.0]<br>*(Cohen's d 0.530)*	0.2 ± 0.6 (2)<br>[0.0, 0.0–2.0]<br>*(Cohen's d 0.734)*	ns (p=0.061)

Data are shown as the mean ± standard deviation of the mean. Number of animals with seizures among all rats in the group (in column "Treatment Group") or total number of seizures recorded during each time epoch is in parentheses. Median and range are shown in brackets. ***Abbreviations***: C d to LEVlow, Cohen's delta to TBILEVhigh treatment group vs TBILEVlow treatment group; h, hour; K-W, Kruskal-Wallis test; LEVlow, levetiracetam 54 mg/kg/d; LEVhigh, levetiracetam 150 mg/kg/d; ns, not significant; TBI, traumatic brain injury; TSA, trichostatin A; Veh, vehicle. ***Statistical significance***: Differences between treatment groups at each time interval were tested using the Kruskal-Wallis test. Differences between the groups were analyzed using Mann-Whitney *U* test: *, p < 0.05; **, p < 0.01 compared with the TBI-Veh group; $^{\#}$, p < 0.05; $^{\#\#}$, p < 0.01 compared with the TBI-TSA group. No differences were found in the TBI-LEVlow group. Time, treatment group, and time x treatment group effects were tested using a general linear model with Bonferroni correction. There were differences in average number of seizures between the treatment groups (p < 0.05. Differences across time intervals (0–24 h, 25–48 h, 49–72 h) within each treatment group were tested using related-samples Friedman's 2-way ANOVA with Bonferroni correction for multiple testing (right column). In each cell, the Cohen's delta between the TBI treatment group *vs.* the TBI vehicle group (in parentheses) showed moderate (≥ 0.50) or large (≥ 0.80) effect sizes.
