# Supplementary material for: Treatment of Status Epilepticus after Traumatic Brain Injury Using an Antiseizure Drug Combined with a Tissue Recovery Enhancer Revealed by Systems Biology
Source: Int J Mol Sci. 2023 Sep 13;24(18):14049. doi: 10.3390/ijms241814049 (PMC10531083; doi:10.3390/ijms241814049)
Supplement: Supplementary file 1 [file ijms-24-14049-s001.zip › ijms-2575599-SI/Supplementary Tables S1- S9/Supplementary Table S7 - Average seizure duration in all rats between 0-72 h post TBI .pdf]

Supplementary Table S7. Average duration (s) of early seizures after traumatic brain injury in different treatment groups between 0–72 h after traumatic brain injury (TBI). Seizure duration is also shown at 24-h epochs (0–24 h, 25–48 h, 49–72 h).

**Treatment Group**	**All seizures** (K-W 0.009)	**Time after TBI (h)**			**Intragroup statistics** (*Friedman's two-way ANOVA*)
		**T1=0–24 h** (K-W 0.075)	**T2=25–48 h** (K-W 0.016)	**T3= 49–72 h** (K-W 0.412)	
TBI-Veh (16)	52 ± 35 (198) [59, 0–126]	43 ± 39 (110) [47, 0–126]	29 ± 35 (57) [10, 0–105]	27 ± 40 (31) [0, 0–119]	ns
TBI-TSA (7)	49 ± 31 (90) [56, 0–87] (*Cohen's d 0.084*)	27 ± 43 (31) [0, 0–100] (*Cohen's d 0.380*)	48 ± 36 (49) [51, 0–109] (*Cohen's d -0.545*)	33 ± 43 (10) [0, 0–100] (*Cohen's d -0.164*)	ns
TBI-LEVlow (10)	23 ± 27 (60) * [16, 0–73] (*Cohen's d 0.891*)	14 ± 30 (28) [0, 0–76] (*Cohen's d 0.796*)	10 ± 17 (18) # [0, 0–42] (*Cohen's d 0.632*)	11 ± 24 (14) [0, 0–69] (*Cohen's d 0.445*)	ns
TBI-LEVhigh (10)	19 ± 27 (10) * [0, 0–66] *(Cohen's d 1.037)* *C d to LEVlow 0.171*	10 ± 22 (2) * [0, 0–66] *(Cohen's d 0.971)* *C d to LEVlow 0.160*	9 ± 14 (5) # [0, 0–32] *(Cohen's d 0.696)* *C d to LEVlow 0.098*	11 ± 25 (3) [0, 0–77] *(Cohen's d 0.453)* *C d to LEVlow 0.016*	ns
TBI-LEVhigh +TSA (10)	12 ± 22 (21) * *, # [0, 0–64] *(Cohen's d 1.298)*	10 ± 17 (16) * [0, 0–47] *(Cohen's d 0.992)*	10 ± 31 (3) # [0, 0–97] *(Cohen's d 0.568)*	9 ± 29 (2) [0, 0–92] *(Cohen's d 0.478)*	ns

Data are shown as the mean ± standard deviation of the mean. Animal numbers or total number of seizures recorded during each time epoch is in parentheses. Median and range are shown in brackets. ***Abbreviations***: C d to LEVlow, Cohen's delta to TBILEVhigh treatment group vs TBILEVlow treatment group; h, hour; K-W, Kruskal-Wallis test; LEVlow, levetiracetam 54 mg/kg/d; LEVhigh, levetiracetam 150 mg/kg/d; ns, not significant; TBI, traumatic brain injury; TSA, trichostatin A; Veh, vehicle. ***Statistical significance***: Differences between treatment groups at each time interval were tested using the Kruskal-Wallis test. Differences between the groups were analyzed using Mann-Whitney *U* test: *, $p < 0.05$; **, $p < 0.01$ compared with the TBI-Veh group; #, $p < 0.05$, compared with the TBI-TSA group. Time, treatment group, and time x treatment group effects were tested using a general linear model with Bonferroni correction. There were differences in average number of seizures between the treatment groups ($p < 0.05$. Differences across the time intervals (0–24 h, 25–48 h, 49–72 h) within each treatment group were tested using related-samples Friedman's 2-way ANOVA with Bonferroni correction for multiple testing (right column). In each cell, the Cohen's delta between the TBI treatment group *vs.* the TBI vehicle group (in parentheses) showed moderate (≥ 0.50) or large (≥ 0.80) effect sizes.
