# Supplementary material for: Treatment of Status Epilepticus after Traumatic Brain Injury Using an Antiseizure Drug Combined with a Tissue Recovery Enhancer Revealed by Systems Biology
Source: Int J Mol Sci. 2023 Sep 13;24(18):14049. doi: 10.3390/ijms241814049 (PMC10531083; doi:10.3390/ijms241814049)
Supplement: Supplementary file 1 [file ijms-24-14049-s001.zip › ijms-2575599-SI/Supplementary Tables S1- S9/Supplementary Table S8 - Average seizure duration in rats with seizures between 0-72 h post TBI .pdf]

Supplementary Table S8. Average seizure duration (s) in each rat with early seizures in different treatment groups between 0–72 h after traumatic brain injury (TBI). Seizure duration is also shown at 24-h epochs (0–24 h, 25–48 h, 49–72 h).

**Treatment Group**	**Average** (K-W 0.406)	**Time after TBI (h)** **T1=0–24 h** (K-W 0.270)	**T2=25–48 h** (K-W 0.149)	**T3= 49–72 h** (K-W 0.561)	**Intragroup statistics (*Friedman's two-way ANOVA*)**
TBI-VEH (13/16)	64 ± 27 (198) [60,19 – 126]	68 ± 24 (110) [64, 43 – 126]	57 ± 27 (57) [61, 19 – 105]	70 ± 31 (31) [69, 34 – 119]	ns
TBI-TSA (6/7)	57 ± 25 (90) [58, 13 – 87] (*Cohen's d 0.255*)	64 ± 44 (31) [77, 15 – 100] (*Cohen's d 0.145*)	56 ± 31 (49) [52, 11 – 109] (*Cohen's d 0.057*)	77 ± 20 (10) [70, 61 – 100] (*Cohen's d -0.233*)	ns
TBI-LEV low (5/10)	45 ± 18 (60) [41, 28 – 73] (*Cohen's d 0.743*)	71 ± 8 (28) [71, 65 – 76] (*Cohen's d -0.099*)	34 ± 7 (18) [31, 28 – 42] (*Cohen's d 0.991*)	55 ± 20 (14) [55, 41 – 69] (*Cohen's d 0.518*)	Unable to compute as too many missing values
TBI-LEV high (4/10)	46 ± 21 (10) [46, 28 – 66] (*Cohen's d 0.690*) *C d to LEVlow -0.044*	50 ± 23 (2) [50, 33 – 66] (*Cohen's d 0.771*) *C d to LEVlow 1.207*	29 ± 4 (5) [29, 25 – 32] (*Cohen's d 1.213*) *C d to LEVlow 0.866*	53 ± 34 (3) [53, 29 – 77] (*Cohen's d 0.547*) *C d to LEVlow 0.072*	ns
TBI-LEV+TSA (3/10)	40 ± 21 (21) [29, 27 – 64] (*Cohen's d 0.922*)	34 ± 11 (16) [29, 27 – 47] (*Cohen's d 1.503*)	97 ± 0 (3) [97, 97 – 97] (*Cohen's d -1.502*)	92 ± 0 (2) [92, 92 – 92] (*Cohen's d -0.694*)	ns

Data are shown as the mean ± standard deviation of the mean. Number of animals with seizures among all rats in the group (in column "Treatment Group") or total number of seizures recorded during each time epoch is in parentheses. Median and range are shown in brackets. ***Abbreviations***: h, hour; K-W, Kruskal-Wallis test; TBI, traumatic brain injury; TBI-LEV low, rats treated with lower dose levetiracetam (54 mg/kg); TBI-LEV high, rats treated with higher dose levetiracetam (150 mg/kg); TBI-LEV+TSA, rats treated with levetiracetam (150 mg/kg) and trichostatin A (1 mg/kg); TBI-TSA, rats treated with trichostatin A; TBI-VEH, rats treated with vehicle. ***Statistical significance***: Differences between treatment groups at each time interval were tested using the Kruskal-Wallis test. Time, treatment group, and time x treatment group effects were tested using a general linear model with Bonferroni correction. Differences across the time intervals (0–24 h, 25–48 h, 49–72 h) within each treatment group were tested using related-samples Friedman's 2-way ANOVA with Bonferroni correction for multiple testing. No differences in the average seizure duration were detected between the treatment groups. Also, there was no change in seizure

duration over the 72-h follow-up. Cohen's delta (in parentheses) between the TBI treatment groups *vs*. the TBI vehicle group, however, showed moderate (≥ 0.50) or large (≥ 0.80) effect sizes of LEV on seizure duration.
