# Supplementary material for: Treatment of Status Epilepticus after Traumatic Brain Injury Using an Antiseizure Drug Combined with a Tissue Recovery Enhancer Revealed by Systems Biology
Source: Int J Mol Sci. 2023 Sep 13;24(18):14049. doi: 10.3390/ijms241814049 (PMC10531083; doi:10.3390/ijms241814049)
Supplement: Supplementary file 1 [file ijms-24-14049-s001.zip › ijms-2575599-SI/Supplementary Tables S1- S9/Supplementary Table S9 - Avergae Racine Score in rats with seizures between 0-72h after TBI.pdf]

Supplementary Table S9. Average Racine score of early seizures in different treatment groups between 0–72 h after traumatic brain injury (TBI). The Racine score is also shown at 24-h epochs (0–24 h, 25–48 h, 49–72 h). Note that values were calculated from animals with early seizures only.

Treatment Group	All seizures (K-W 0.070)	Time after TBI (h)			Intragroup statistics (*Friedman's two-way ANOVA*)
		**T1=0–24 h** (K-W 0.613)	**T2=25–48 h** (K-W 0.075)	**T3= 49–72 h** (K-W 0.501)	
TBI-Veh (11/16)	0.20 ± 0.26<br>[0.00, 0.00 – 0.63]	0.04 ± 0.07<br>[0.00, 0.00 – 0.20]	0.74 ± 0.72<br>[0.74, 0.00 – 2.00]	0.46 ± 0.55<br>[0.24, 0.00 – 1.29]	ns
TBI-TSA (6/7)	0.42 ± 0.29<br>[0.40, 0.00 – 0.90]<br>(*Cohen's d* -0.816)	0.05 ± 0.04<br>[0.06, 0.00 – 0.08]<br>(*Cohen's d* -0.102)	0.64 ± 0.20<br>[0.64, 0.40 – 0.85]<br>(*Cohen's d* 0.176)	0.83 ± 0.76<br>[1.00, 0.00 – 1.50]<br>(*Cohen's d* -0.604)	ns
TBI-LEVlow (4/10)	0.62 ± 0.48<br>[0.74, 0.00 – 1.00]<br>(*Cohen's d* -1.295)	0.32 ± 0.45<br>[0.32, 0.00 – 0.64]<br>(*Cohen's d* -1.701)	1.22 ± 0.31<br>[1.22, 1.00 – 1.44]<br>(*Cohen's d* -0.706)	0.59 ± 0.83<br>[0.59, 0.00 – 1.17]<br>(*Cohen's d* -0.207)	Unable to compute
TBI-LEVhigh (3/10)	0.00 ± 0.00<br>[0.00, 0.00 – 0.00]<br>*(Cohen's d 0.867)*<br>*C d to LEVlow 1.666*	0.00 ± 0.00<br>[0.00, 0.00 – 0.00]<br>*(Cohen's d 0.571)*<br>*C d to LEVlow 0.707*	0.00 ± 0.00<br>[0.00, 0.00 – 0.00]<br>*(Cohen's d 1.200)*<br>*C d to LEVlow 6.792*	0.00 ± 0.00<br>[0.00, 0.00 – 0.00]<br>*(Cohen's d 0.918)*<br>*C d to LEVlow 1.000*	ns
TBI-LEVhigh +TSA (2/10)	0.00 ± 0.00<br>[0.00, 0.00 – 0.00]<br>*(Cohen's d 0.830)*	0.00 ± 0.00<br>[0.00, 0.00 – 0.00]<br>*(Cohen's d 0.606)*	0.00 ± 0.00<br>[0.00, 0.00 – 0.00]<br>*(Cohen's d 1.039)*	0.00 ± 0.00<br>[0.00, 0.00 – 0.00]<br>*(Cohen's d 0.838)*	ns

Data are shown as the mean ± standard deviation of the mean. Number of animals with assigned Racine score, of all rats in the group (in column "Treatment Group"), is in parentheses. Median and range are shown in brackets. ***Abbreviations***: C d to LEVlow, Cohen's delta to TBILEVhigh treatment group vs TBILEVlow treatment group; h, hour; K-W, Kruskal-Wallis test; TBI, traumatic brain injury; TBI-LEV low, rats treated with lower dose levetiracetam (54 mg/kg); TBI-LEV high, rats treated with higher dose levetiracetam (150 mg/kg); TBI-LEV+TSA, rats treated with levetiracetam (150 mg/kg) and trichostatin A (1 mg/kg); TBI-TSA, rats treated with trichostatin A; TBI-VEH, rats treated with vehicle. ***Statistical significance***: Differences between treatment groups at each time interval were tested using the Kruskal-Wallis test. Time, treatment group, and time x treatment group effects were tested using a general linear model with Bonferroni correction. There were differences in average Racine score between the treatment groups ($p < 0.05$). Differences across the time intervals (0–24 h, 25–48 h, 49–72 h) within each treatment group were tested using related-samples Friedman's 2-way ANOVA with Bonferroni correction for multiple testing. The Racine score showed no change over the 72-h follow-up. Cohen's delta (in parentheses) between the TBI treatment groups *vs*. the TBI vehicle group showed moderate (≥ 0.50) or large (≥ 0.80) effect sizes of LEV on seizure duration.
